# Recent Progress in Interfacial Dipole Engineering for Perovskite Solar Cells

**DOI:** 10.1007/s40820-023-01131-4

**Published:** 2023-07-07

**Authors:** Yinyi Ma, Jue Gong, Peng Zeng, Mingzhen Liu

**Affiliations:** 1 School of Materials and Energy, University of Electronic Science and Technology of China, Chengdu, 611731 People’s Republic of China; 2 State Key Laboratory Electronic Thin Film and Integrated Devices, University of Electronic Science and Technology of China, Chengdu, 611731 People’s Republic of China

**Keywords:** Perovskite solar cells, Interfacial dipoles, Analytical techniques

## Abstract

Design and modification of interfaces have been the main strategies in developing perovskite solar cells (PSCs). Among the interfacial treatments, dipole molecules have emerged as a practical approach to improve the efficiency and stability of PSCs due to their unique and versatile abilities to control the interfacial properties. Despite extensive applications in conventional semiconductors, working principles and design of interfacial dipoles in the performance/stability enhancement of PSCs are lacking an insightful elucidation. In this review, we first discuss the fundamental properties of electric dipoles and the specific roles of interfacial dipoles in PSCs. Then we systematically summarize the recent progress of dipole materials in several key interfaces to achieve efficient and stable PSCs. In addition to such discussions, we also dive into reliable analytical techniques to support the characterization of interfacial dipoles in PSCs. Finally, we highlight future directions and potential avenues for research in the development of dipolar materials through tailored molecular designs. Our review sheds light on the importance of continued efforts in this exciting emerging field, which holds great potential for the development of high-performance and stable PSCs as commercially demanded.

## Highlights

- The fundamental properties of electric dipoles and their specific roles in perovskite solar cells are discussed.
- Research progress of interfacial dipoles in perovskite solar cells is summarized.
- Challenges of deterministic characterization of electric dipoles and future perspectives are highlighted.

## Graphical abstract

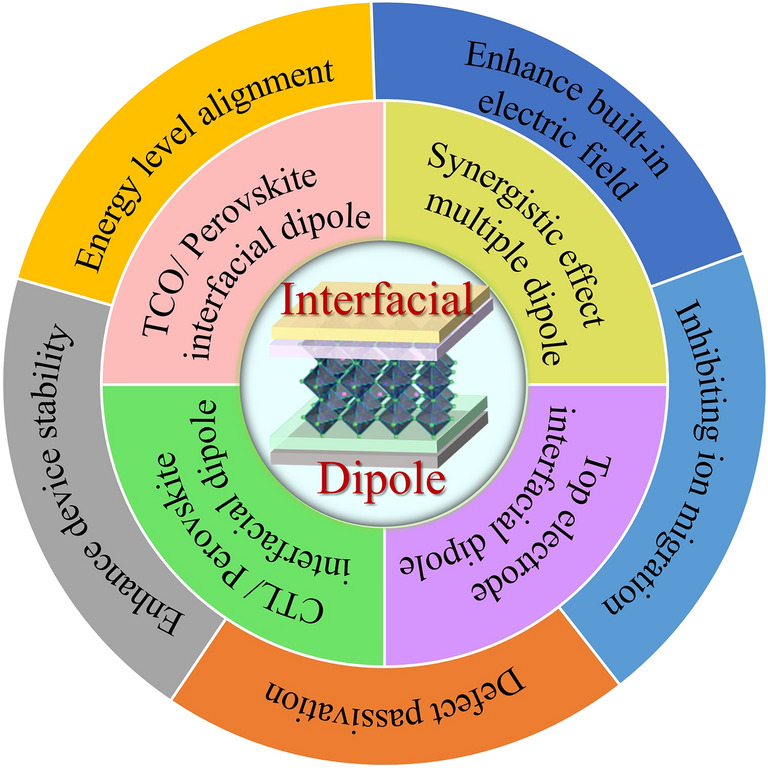

## Introduction

In recent years, organic–inorganic lead halide perovskites have shown great potential for solar cell applications [1–4]. The power conversion efficiency (PCE) of perovskite solar cells (PSCs) has rapidly surged in the past decade, reaching a certified 25.7% nowadays, which is comparable to the conversion efficiency of crystalline silicon technology [5, 6]. The rapid development of PSCs can be attributed to their superior optoelectronic properties, such as excellent optical absorption coefficient (10^5^ cm^−1^), long carrier diffusion lengths (> 1 μm), tunable direct bandgaps, and unusual defect tolerance [7]. In particular, an advantage that should not be overlooked is the facile and inexpensive manufacturing process with solution-processable fabrication [8, 9]. In addition to this strong point, it also benefits from the lessons quickly learned from other photovoltaic (PV) technologies, such as dye-sensitized solar cells (DSSCs), organic photovoltaics cells (OPVs) and silicon solar cells [10].

However, optoelectronic performance of PSCs is limited by the presence of inherent defects such as traps, vacancies, and uncoordinated ions at the perovskite grain boundary or device interfaces [11]. These defects cause severe nonradiative carrier recombination, thereby reducing the final PCE and operational stability of the devices [12]. In particular, the number of defects at the surface of perovskite thin films is found to be two orders of magnitude higher than in the bulk, and most of these defects are deep energy level traps [13]. Moreover, energy level mismatch at the interface increases carrier recombination losses, severely limiting the overall efficiency of PSCs [14]. A retrospective look at the development of silicon solar cells enables the establishment of field-effect passivation (FEP) in light of the AlO_X_-passivated backside cells [15–17]. In detail, FEP gives rise to the generation of an interfacial dipole electric field through the insertion of a dielectric film that avoids recombination regions by repelling and separating free charges at the interface [18]. The effect of FEP is essential to the reduction of recombination velocity (kinetic process), which provides an important guide for perovskite-based devices, and FEP has recently been widely proposed for the optimization of PSCs devices [19]. Similarly, the use of a cathode interlayer material poly[(9,9-bis(3′-(N,N-dimethylamino)propyl)-2,7-fluorene)-alt-2,7-(9,9-dioctylfluorene)] (PFN) in OPVs was a key factor in the significant breakthrough of device PCE [20, 21]. The strong electric dipole moment of PFN, consistent with the built-in electric field, promotes efficient carrier separation and selective transport. This wealth of experience from other PV fields has been applied to PSCs, contributing to their rapid development [22].

**Fig. 1:**
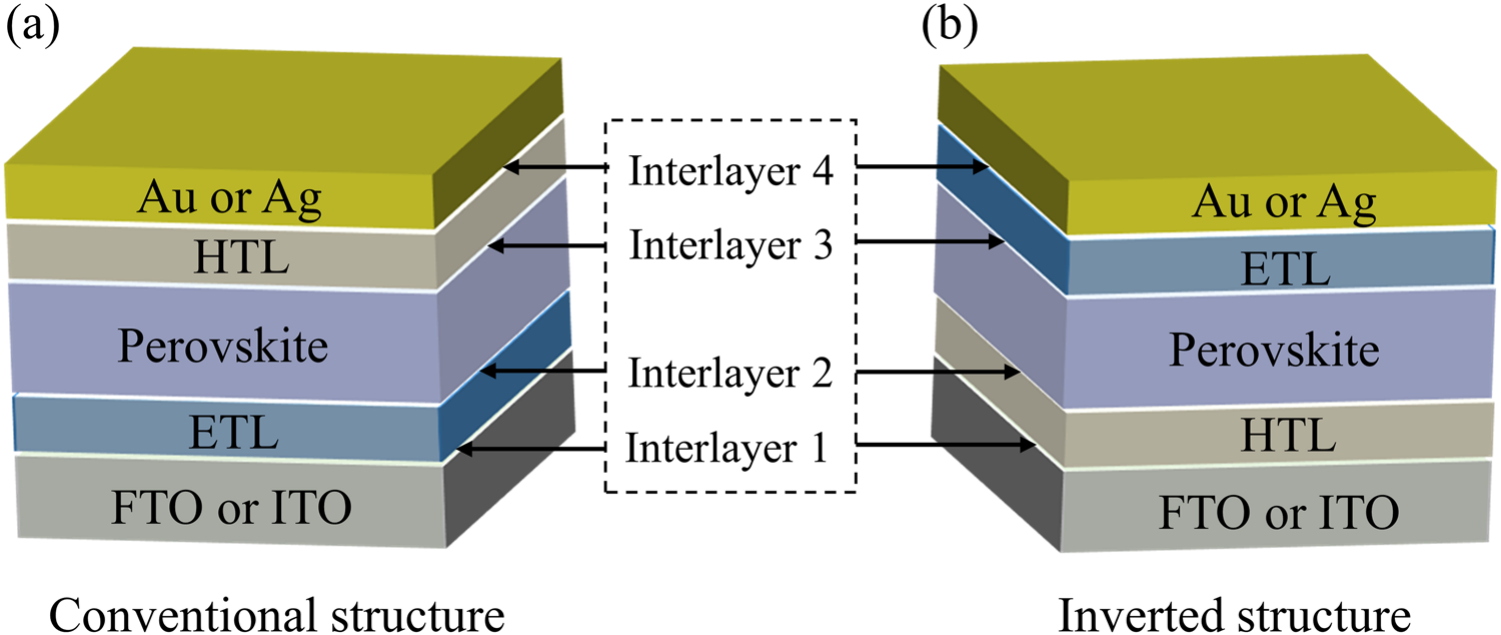
Schematic illustration of functional layer and interfaces in PSCs. **a** Conventional n-i-p device structure and **b** inverted p-i-n structure

Architecture of PSCs is also critical to their efficiency, as the morphology of the different layers and their interfacial properties are crucial to the carrier generation and extraction [23]. PSCs are usually formed by stacking anode/cathode, carrier transport layers (electron/hole transport layers, denoted ETLs/HTLs, respectively) and perovskite active layers, thus forming a multi-interface system [24]. The optoelectronic properties of these interfaces (Fig. 1), such as (i) electrode/ETL, (ii) ETL/perovskite, (iii) perovskite/HTL and (iv) HTL/electrode, not only affect the device performance but also the operational stability [25]. Among them, interfacial defects, which mostly have deep energy level, still significantly limit the efficiency improvement of PSCs [26]. In recent years, attentions have been paid to the incorporation of interlayers between the perovskite active layers and the electrodes, which can significantly improve the device performance [27]. Among them, interfacial dipole modification is a relatively simple and effective strategy to improve device efficiency [28, 29]. As mentioned earlier, although interfacial dipoles have been widely used in other PV applications such as silicon solar cells [15–17]and OPVs [20, 21], the application in PSCs is still at a beginning stage [30]. The functionality of dipole interlayers involves various mechanisms, such as causing a larger electron/hole quasi-Fermi level splitting to significantly enhance the built-in electric field, thereby promoting directional transport of charge carriers, avoiding Fermi level pinning, and regulating the interfacial energy level offset [31, 32]. As such, working principles of the mostly adopted dipole materials in PSCs are still unclear. Along with few literatures available that focuses on dipole-modulated PSCs, optimal designs and upscale applications of dipole materials face austere limitations [28].

In this review, we outline recent advances in improving the efficiency and stability of PSCs based on the interfacial dipole strategies. We aim to highlight the mechanisms of dipole formations and the controlling mechanism of device performance in PSCs, along with the corresponding characterization techniques used to detect and probe the interfacial dipole layers. In specific, we introduce a number of characterization techniques to reveal the microscopic configurations of dipole molecules at the interface. For example, sum-frequency spectroscopy (SFG) based on second-order nonlinear optical techniques and near-edge X-ray absorption fine-structure spectroscopy (NEXAFS) are employed to reveal orientational configuration of the dipoles. Finally, we provide perspectives and insights into the rational design of promising interfacial dipole materials.

## Effects of Interfacial Dipoles in PSCs

### Field-effect Properties of Electric Dipoles

**Fig. 2:**
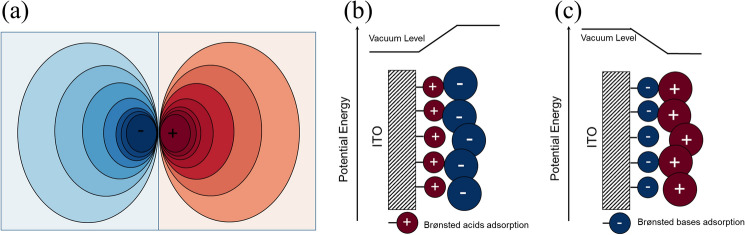
Depiction of electric dipole and its effects in band bending. **a** Electrostatic potential contour plot of a single electric dipole. Schematic illustration of the vacuum horizontal displacement of the ITO surface modified by Brønsted-Lowry **b** acids and **c** bases

From chemistry standpoint, a dipole occurs when the partial positive and negative charges between two covalently bonded atoms or charge-carrying atoms/groups are spatially separated from each other within a molecule (e.g., zwitterions) [33]. The formation of a dipole depends on the polarity of the bond, which is determined by the bond is determined by the difference in electronegativity between the two atoms involved [34]. Note that dipoles, whether electrical or magnetic, can be quantified by their dipole moment (μ). Where the electric dipole moment is the distance d between charges multiplied by the amount of charge q. The unit of dipole moment is Debye, with one Debye being 3.34 × 10^–30^ C m [35]. A dipole moment is a vector that has both magnitude and direction. The direction of the electric dipole moment is from the negative charge to the positive charge. Figure 2a shows the electrostatic potential contour plot of a horizontally oriented electric dipole with infinitely small size [28].

An interfacial dipole layer in PSCs can be approximated as consisting of many single electric dipoles arranged in order [36]. The dipole interlayer can change the work functions of conductive electrodes and affect the carrier transport across the interface [37]. Figure 2b, c illustrates one of the examples, where the use of acids and bases to treat electrode materials. The acidic condition protonates the ITO surface, forming an interfacial dipole layer oriented away from the electrode, which modifies and increases the work function of ITO. Alkaline conditions form an interfacial dipole layer pointing to the electrode and reduce the work function of ITO [38].

#### Modified Work Function to Match Energy Levels

The band shift at the interface is critical to the charge transfer and recombination processes, which in turn affects the device PCE [39]. If the interfacial energy levels are matched, it is favorable for electron to transfer from conduction band minimum (CBM) of perovskite layer to the lowest unoccupied molecular orbital (LUMO) of organic ETL or CBM of inorganic ETL, and finally to the cathode. Similarly, holes will transfer in the opposite direction. To achieve high-concentration carrier extraction, an excellent ohmic contact between the interface layers is required [40].

In intrinsic or non-degenerate doped semiconductors, vacuum level (*E*_vac_) is defined as the energy at which electrons can escape from solid into the vacuum. *E*_vac_ defines a partial vacuum level rather than an absolute vacuum level [41]. Thus, an electric dipole operating on a locally horizontal surface can deviate from the vacuum level, where position of the E_vac_ can be influenced by the surface treatment [42]. Molecular insertion with a permanent dipole moment is expected to tune the energy level and reduce the energy gap difference, further reducing or preventing the occurrence of unwanted charge recombination at the interface. According to Eq. (1), where the offset of work function (∆φ) can be modulated by the magnitude and direction of the surface-adsorbed dipole moment [43]:

1 $$ \Delta \varphi = - N\left[ {\frac{{\mu_{ \bot } }}{{\varepsilon_{0} k}} + \frac{{\mu_{M - S} }}{{\varepsilon_{0} k_{M - S} }}} \right] $$

where *N* represents the adsorption density, *ε*_0_ and *k* the permittivity of free space and the dielectric constant of the adsorbate layer, respectively; $\mu_{ \bot }$ is the dipole moment perpendicular to the surface. µ_*M*−*S*_ is the effective dipole for interfacial charge-transfer interaction, and *k*_*M*−*S*_ is the dielectric constant component of the interfacial interaction.

Self-assembled monolayers (SAMs) are densely arranged and ordered two-dimensional dipole materials on the surface that strongly influence the energy level alignment by imparting dipole moments at the interface [44–48]. SAMs have two adsorption modes on the material surfaces, namely, physisorption and chemisorption; while from the structural standpoint, SAMs are composed of anchoring group, linker/spacer, and terminal functional group [49, 50]. Calculations show that the groups at both ends of the SAMs are electrostatically decoupled, and depolarization is observed in the interface [51, 52]. Conjugated polymers represented by PFN are an important milestone in the development of OPVs [20], in which strong interfacial dipoles play an important role in improving device performance, highlighting the advantages of interfacial dipole layers. In addition, neutral molecules or electrolytes, zwitterion-based molecules, and electrolyte graft copolymers are also commonly used dipole interface materials [30].

#### Enhanced Built-in Electric Fields at Interfaces

According to the semiconductor thermal equilibrium theory and Anderson model, when two different semiconductors are in contact, a unified Fermi energy level will be generated due to the diffusion of carriers, resulting in band bending [53]. This diffusion and drift create a built-in electric field with a concomitant space charge depletion region. The role of the built-in electric field is to separate free charges in the depletion region, and the built-in electric field is generated by the difference in work functions of ETL to HTL upon the fabrication of PSCs [54]. *V*_OC_ originates from the splitting of holes and electrons at the quasi-Fermi level [55], and the change in *V*_OC_ can be estimated by Eq. (2):

2 $$ V_{OC} = E_{{F_{n} }} - E_{{F_{p} }} = E_{G} + kT \cdot \ln \left( {\frac{n}{{N_{C} }}} \right) + kT \cdot \ln \left( {\frac{n}{{N_{V} }}} \right) $$

where *E*_Fn_ and *E*_Fp_ are the quasi-Fermi levels of electrons and holes, with *n* and *p* the electron and hole densities, respectively; *N*_C_ and *N*_V_ are the effective densities of states in conduction and valence bands, respectively; and *kT* is the thermal energy. The configuration has a vertical dipole moment layer parallel to the built-in electric field, allowing easy tuning of the actual built-in electric field of the device. The built-in electric field increases or decreases in the final device according to the dipole electric field and the built-in electric field [56]. On the other hand, increment of the built-in electric field can significantly enhance the quasi-Fermi level splitting of holes or electrons, thereby affecting the device's *V*_OC_ [57]. This method of increasing the built-in electric field and *V*_OC_ of the device by adding an interfacial dipole layer does not directly change the bandgap of the active material. It preserves the range of the solar spectrum available to active materials without sacrificing light absorption [30].

#### Enhancing Interfacial Charge Carrier Dynamics

Solar cells can be simplified to a physical model consisting of a carrier source and two boundaries [53]. The perovskite active layer is excited by light to generate free carriers, and the interface determines the distribution and transport of carriers. This process can be described by the one-dimension steady-state diffusion Eq. (3) [53]:

3 $$ D_{n} \frac{{\partial^{2} n\left( x \right)}}{{\partial x^{2} }} + G_{n} \left( x \right) - \frac{{n - n_{0} }}{{\tau_{n} }} = 0 $$

where *D*_*n*_ is the carrier diffusion coefficient of the semiconductor, *G*_*n*_(x) the generation rate of carriers, *n*_*0*_ the carrier density at thermal equilibrium, and *τ*_*n*_ the carrier lifetime. Among them, the free carriers located in the depletion region the boundary condition for sub-transport can be expressed in terms of current density, i.e., where σ is the conductivity and *E* is the electric field strength. Therefore, charge transport in solar cells is determined by carrier distribution and electric field. The carrier distribution and transport in PSCs are more complex than the ideal model but still benefit from the above-mentioned basic principles [58, 59]. The carrier dynamics at the interface of the perovskite layer and the carrier transport layer affect the conversion efficiency of the device through aspects of charge generation, charge transfer, charge extraction, and charge recombination [60].

### Physicochemical Function of Electric Dipoles

Although the exciton binding energy of perovskite is low, which suggests that these excitons can be dissociated to generate free carriers at room temperature [61, 62]. The energy level arrangement at the interface determines the charge transfer/transport and is related to the recombination at the interface, and thus greatly affects the device performance [63]. As with other types of PV cells, there is still a large probability of interfacial recombination of free carriers when they encounter selective contact at the interface [64]. Considering the Shockley-Queisser efficiency limit (33.7%) of single-junction PSCs [65–68], PCE of PSCs still has room of improvement. Interface engineering is an effective way to adjust the interface properties to overcome the interfacial loss without destroying the lower layer properties. The optimization of the interface can also protect the device from degradation and improve its stability of the device. Previous studies have demonstrated an elegant alternative to enhance the performance of PSCs by inserting interfacial dipole formulas [19, 28]. The interfacial dipole layer can enhance the performance of PSCs in various ways, thus involving various mechanisms of action. In addition to careful selection of the appropriate dipole molecular structures, the surface stacking state of the dipole molecule also needs to be considered [69]. A disorderly orientation of the dipole moment will lead to a reduction or even cancellation of the net dipole moment. By strictly controlling the dipole direction, potential negative impacts to the PV parameters, such as loss in open circuit voltage (*V*_OC_), can be mitigated. The functionality and importance of interfacial dipoles are discussed in detail in the following sections.

#### Passivating Interfacial Defects

In a typical PSCs structure, dangling bonds that may exist on all interfaces will generate trap states at the interfaces, which will lead to charge recombination accumulation, recombination loss, and hysteresis, which will further reduce the PCE [70]. Huang et al. [71] reported the spatial and energy distribution of trap states in PSCs, confirming that the charge trap density at all interfaces of polycrystalline perovskite films is one to two orders of magnitude larger than that in the film interior. Therefore, the interface is a key step that determines the efficient transport of photogenerated carriers. Surface trap states, such as electronic coupling and chemical bonding, can be terminated at the interface by forming chemical interactions, which can be collectively referred to as chemical passivation [72, 73]. For example, Ruan et al. [74] reported the use of Pb^2+^ cations with insufficient Cl^−^ within the polar molecule 4-chlorobenzoic acid (4-CLBA) to form a passivation effect. The defect density of perovskite films is significantly reduced.

Furthermore, inspired by the formation of strong interfacial dipoles by intercalating dielectric films in silicon cells, FEP as caused by the dipole has also been developed in PSCs in recent years [19, 75]. By tuning the direction of charge polarization, charge separation can be significantly enhanced, thereby mitigating charge recombination at the interface. This method provides a more comprehensive passivation effect than chemical bonding passivation. Both mechanisms may coexist in most passivation scenarios of ammonium salts and other interfacial dipoles [76]. This requires further research and discussion in our community.

#### Suppressing Ion Migration Across Device Interface

As a material with both semiconductor and ionic conductor properties, perovskite is characterized by the migration and accumulation of charged ions at the interface, which directly leads to local crystal structure changes, which severely decompose perovskite films [77]. On the other hand, ion migration is one of the sources of hysteresis in PSCs. Snaith et al. [78] used a numerical drift–diffusion model to test the conjecture that ion migration causes J-V hysteresis. The ion transport pathways in PSCs devices are divided into two categories, (1) ion exchange and ion penetration between perovskite and charge transport layers on both sides, and (2) the diffusion of mobile ions in the metal electrode from the electrode to the charge transport layer.

It is generally believed that defects provide a path for the migration of charged ions, and interfacial dipole molecules can provide effective defect passivation to alleviate the ion migration effect. Not long ago, Li et al. [79] reported the insertion of L-phenylalanine (PAA) dipole molecules at the interface between ETL and perovskite, which effectively suppressed iodide migration in perovskite films through an enhanced bulk passivation strategy. Wang et al. [80] recently developed organic–inorganic (OI) complex dipolar molecular materials for interfacial processing. Among them, the strong interaction between OI complexes and perovskite can suppress ion migration, and the dipole moment induced by OI complexes inhibits ion migration from HTL to the perovskite layer.

#### Enhancing Moisture/Oxygen Resistance at Device Interface

To date, the greatest obstacle encountered in the commercialization of PSCs is still the stability issue [81]. The stability problem is not only closely related to the intrinsic properties of the materials of each layer but also depends on the degraded interface inside the device. Because the interface is the weakest link, degradation occurs preferentially at the surface interface [53]. For example, some studies have shown that inorganic ZnO_2_ or SnO_2_ electron transport layers can decompose perovskites [82]. In particular, the sensitivity of PSCs to humidity and temperature makes a commercial application more difficult. Snaith et al. [83] reported that if PSCs do not provide additional protection, a series of conventional hole transport materials undergo significant degradation in air at 80 ℃ in only a few hours. In turn, the perovskite component is decomposed to generate yellow PbI_2_. Although some researchers have also developed some highly stable transport materials, in practice, introducing a hydrophobic interlayer is a simpler way to achieve similar protective effects [84–86]. Numerous research reports have demonstrated that the intercalation of interfacial dipole materials not only improve the PV performance, but also enhance the stability of PSCs [19, 29, 87, 88]. This can be attributed to defect passivation and functionality of the end groups of dipole molecules, such as organic molecules with hydrophobic end groups. Increased resistance to moisture and hygroscopic dopants thus signifies the promise of long-term stability of unencapsulated PSCs [29, 89].

## Site-specific Interfacial Dipole Effects in PSCs

As mentioned earlier, efficient PSCs are usually sandwich structures with multiple different interfaces [23, 24]. Interfaces are of overwhelming importance in all thin-film devices [38]. The interfacial dipole plays a key role in affecting charge dynamics, defect passivation, and device stability enhancements in PSCs. This section summarizes the latest research progress on interfacial dipole materials in PSCs, focusing on the confirmation of the direction and strength of the interfacial electric dipole moment, elaborating the working mechanism of dipole materials at various interfaces in PSCs, as well as dipole materials impact on device performance.

### Bottom Transparent Conductive Oxides (TCOs)/Perovskite Active Layer

Fluorine-doped tin oxide (FTO) and indium tin oxide (ITO) are the most commonly used bottom transparent oxides (TCOs) in PSCs [90]. To facilitate the commercialization of PSCs, it is highly desirable to develop ETL- or HTL-free device structures that are cost-effective and have simpler fabrication processes [91, 92]. However, direct contact between the conductive electrode and the perovskite active layer is not desirable due to the energy level mismatch with the adjacent layers, which leads to poor carrier extraction and loss of efficiency [93]. Previous studies have shown that although the work function of TCOs may depend on the cleaning method [94], the Fermi level of TCOs is close to the bandgap center of the perovskite layer, resulting in an apparent work function difference. At the device level, this mismatch is amplified and requires modulation of the TCOs work function to match that of the perovskite layer. One simple way to achieve this is by introducing a dipole material at the interface.

**Fig. 3:**
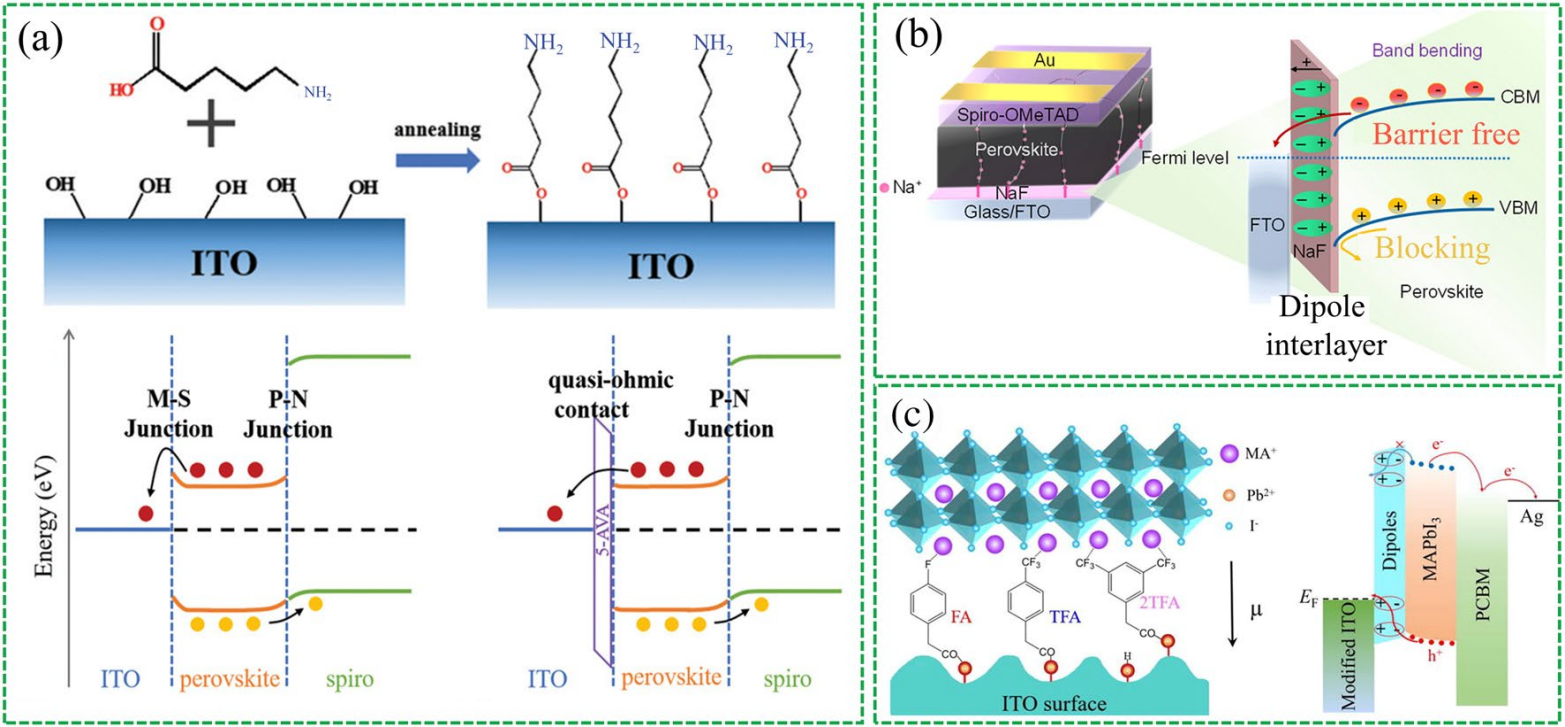
Buried bottom interfacial dipole in CTL-free device. **a** Schematic diagram of the self-assembly of 5-AVA on ITO and the energy level arrangement of the interface. Reproduced with permission from Ref. [95]. Copyright 2021, John Wiley & Sons. **b** Schematic illustration of band bending caused by introducing inorganic compound NaF as interfacial dipole. Reproduced with permission from Ref. [96]. Copyright 2022, American Chemical Society. **c** Introducing three different dipole interlayers in HTL-free PSCs in an inverted structure to tune the surface work function of ITO substrates. Reproduced with permission from Ref. [99]. Copyright 2022, Elsevier

For example, in normal structure without ETL device, it is necessary to effectively reduce the work function of TCOs [91, 93]. Various dipole materials have been explored for this purpose. For instance. Zhu et al. [95] inserted 5-aminovaleric acid (5-AVA) into the ITO/perovskite interface to form an insulating dipole layer, which effectively reduced the apparent work function of ITO by 0.58 eV, resulting in a quasi-ohmic contact (Fig. 3a). In addition, inorganic compounds have also been discussed. Akin et al. [96] introduced sodium fluoride (NaF) as an interfacial layer in ETL-free PSCs (Fig. 3b), which not only reduced the work function of FTO by forming an interfacial dipole but also facilitated crystallite enlargement and spontaneous passivation because of some Na^+^ ions could migrate into the absorber layer. Recently, ionic liquids have also been used to modify the surface of TCOs. Chen et al. [97] found that methylammonium acetate (MAAc) could be physisorbed on the ITO electrode, constructing an interfacial dipole layer in situ that facilitated charge transport, leading to the development of ETL-free PSCs with a high conversion efficiency of 21.08%.

In inverted PSCs, increasing the surface WF of TCOs is essential to promote hole transport at the buried interface. One approach is to introduce a dipole molecule directed towards the TCOs side of the dipole moment [98]. Yu et al. [99] demonstrated the use of three different dipole interlayers, namely 4-Fluorophenylacetic acid (FA), 4-(Trifluoromethyl) phenylacetic acid (TFA), and 3,5-Bis(trifluoromethyl) phenylacetic acid (2TFA). The carboxyl groups in these dipole materials passivate the surface terminal -OH groups on the ITO surface (Fig. 3c) and change the wettability of the ITO substrate, resulting in perovskite films with fewer defect states. The researchers claimed that 2TFA achieved the largest PCE of 20.19% due to its largest molecular dipole moment.

**Fig. 4:**
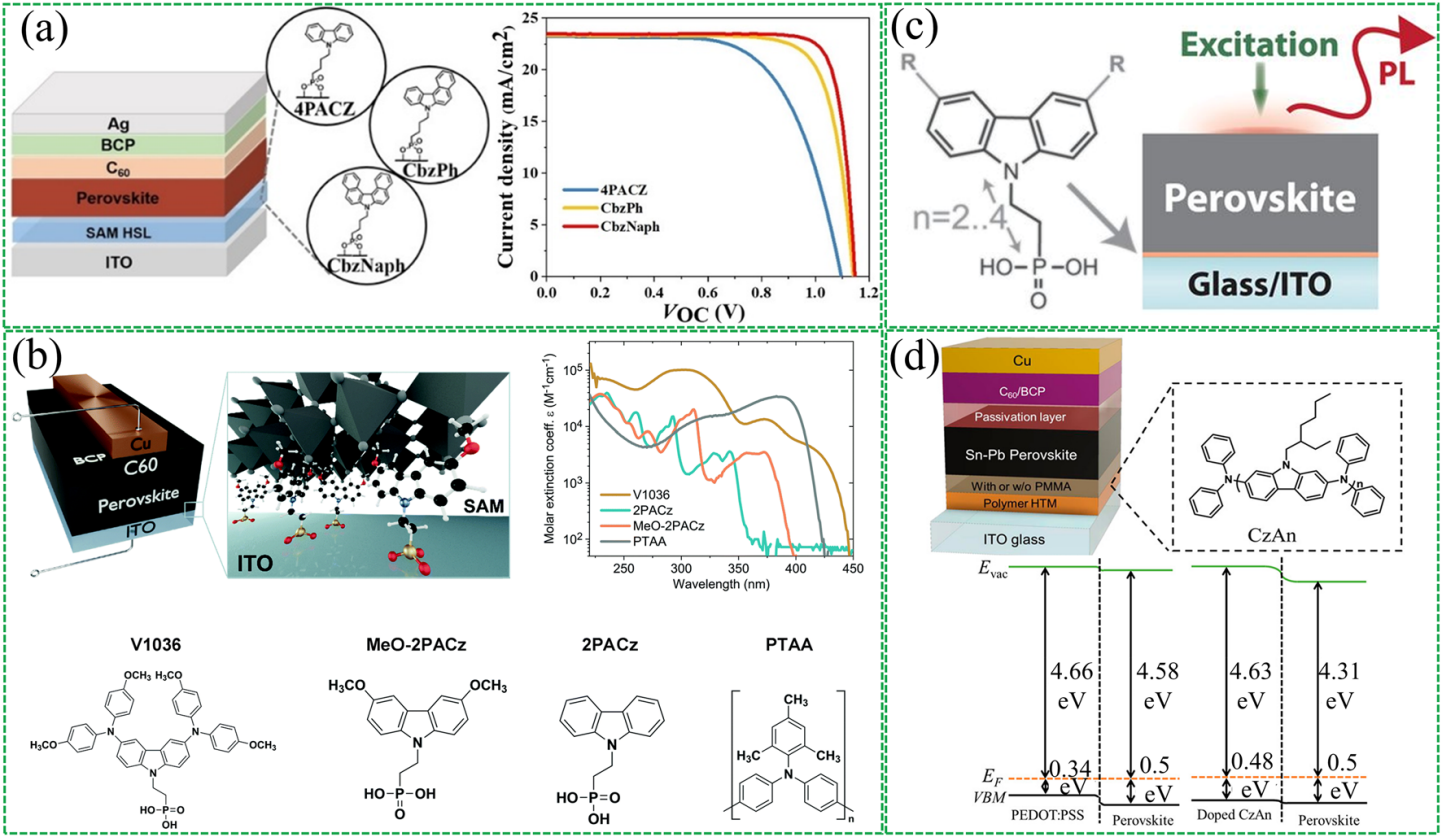
Interfacial dipole modified bottom TCOs based on SAMs. **a** Device architecture of the inverted PSCs and current density–voltage (*J–V*) characteristics. Reproduced with permission from Ref. [101]. Copyright 2022, John Wiley and Sons. **b** Solar cell device architecture and molecule structures investigated in this work. Reproduced with permission from Ref. [102]. Copyright 2019, Royal Society of Chemistry. **c** Photoluminescence properties and stability assessment of perovskite films on different substrates. Reproduced with permission from Ref. [103]. Copyright 2020, American Association for the Advancement of Science. **d** Device structure of Sn–Pb PSCs and molecular structure of CzAn. Reproduced with permission from Ref. [104]. Copyright 2022, Elsevier

SAMs have demonstrated remarkable enhancement efficacy in inverted-structure PSCs. SAMs not only significantly reduce material consumption and parasitic absorption but also possess various substrate compatibilities, simpler non-invasive schemes, and green solvent processability. Among these properties, the most promising are carbazole-derived SAMs with rich electron characteristics that render them excellent hole-selective materials. However, such SAMs typically have small dipole moments due to carbazole's high symmetry and planar structure, which hinders their effective adjustment of the work function of ITO substrate, limiting the photovoltage that devices output. Albrecht's group [100] used a new generation of carbazole-based SAMs to replace poly(triaryl amine) (PTAA) and adjust the work function of the ITO substrate. The SAMs with phosphonic-acid anchoring groups facilitate the ohmic transport of charge carriers at the electrode/perovskite interface. Jiang et al. [101] reported a molecular design (Fig. 4a) based on which two types of carbazole-based SAMs, CbzPh and CbzNaph were synthesized through asymmetric and helical π-expansion engineering, respectively, with both resulting in enhanced dipole moments and π-π interactions. CbzNaph had the largest molecular dipole moment (2.41 D) and compact π-π stacking (π-π distance = 3.34 Å), forming the densest assembly and suitable band alignment, ultimately achieving a top PCE of 24.1% on champion PSC devices. Albrecht et al. [102] reported two new hole-selective contact materials (Fig. 4b), MeO-2PACz ([2-(3,6-dimethoxy-9H-carbazol-9-yl)ethyl]phosphonic acid) and 2PACz ([2-(9H-carbazol-9-yl)ethyl]phosphonic acid), both of which have carbazole and phosphonic acid anchoring groups that can form SAMs on substrates. It was found that both SAMs exhibited greater hole selectivity than PTAA, and the difference in work function between these two molecules was attributed to the difference in molecular dipole moment of the hole-selective segment. 2PACz has a larger molecular dipole moment, which aligned with the VBM of the perovskite, ultimately exhibiting the best device efficiency. In another study by Albrecht et al. [103], Me-4PACz ([4-(3,6-dimethyl-9H-carbazol-9-yl)butyl]phosphonic acid) was designed as a hole-selective layer in perovskite solar cells (Fig. 4c). The perovskite/silicon tandem device achieved a certified PCE of 29.15% due to the rapid hole extraction and minimization of non-radiative recombination at the hole-selective interface of this novel SAMs. Furthermore, Me-4PACz had a rapid hole extraction and effective passivation effect at the hole-selective interface, slowing down the photo-induced halide segregation of 1.68 eV wide-bandgap perovskite. In addition, Huang et al. [104] reported a hole transport material based on carbazole-based polymer (Fig. 4d), poly[(phenyl)imino[9-(2-ethylhexyl)carbazole]-2,7-diyl] (CzAn). It was found that CzAn had a low intrinsic hole conductivity, but with doping and surface modification, perovskite thin film prepared on the CzAn hole transport material exhibited greater crystallinity, lower trap density, and larger carrier mobility. Finally, a PCE of 22.6% was achieved in tin–lead PSCs, much higher than the devices based on conventional PEDOT:PSS HTL.

### Top Electrode/Perovskite Active Layer

The junction interface between perovskite and electrode materials is more complex than that of the inorganic semiconductor interface due to the ionic nature and high reactivity of perovskite [105]. The widely observed problem of poor stability remains the primary reason hindering the commercialization of PSCs. various factors affect the interaction between highly reactive perovskites and electrodes. For instance, water and oxygen trap electrons from the perovskite layer, resulting in p-type doping at the interface [63, 106]. Common metal electrodes with low work functions such as silver (Ag) and aluminum (Al) corrode and undergo significant degradation when in contact with perovskites [107, 108]. Even inert gold (Au) still reacts with perovskites [109]. To address this issue, many efforts have been made to investigate perovskite interfaces in the hope of suppressing chemical reactions and interfacial defects to ultimately achieve ideal contacts. The insertion of dipole materials at the top electrode and perovskite layer not only changes the dynamic of charge carriers on the interface and promotes charge carrier transfer, but also prevents interfacial chemical reactions and enhances device stability.

**Fig. 5:**
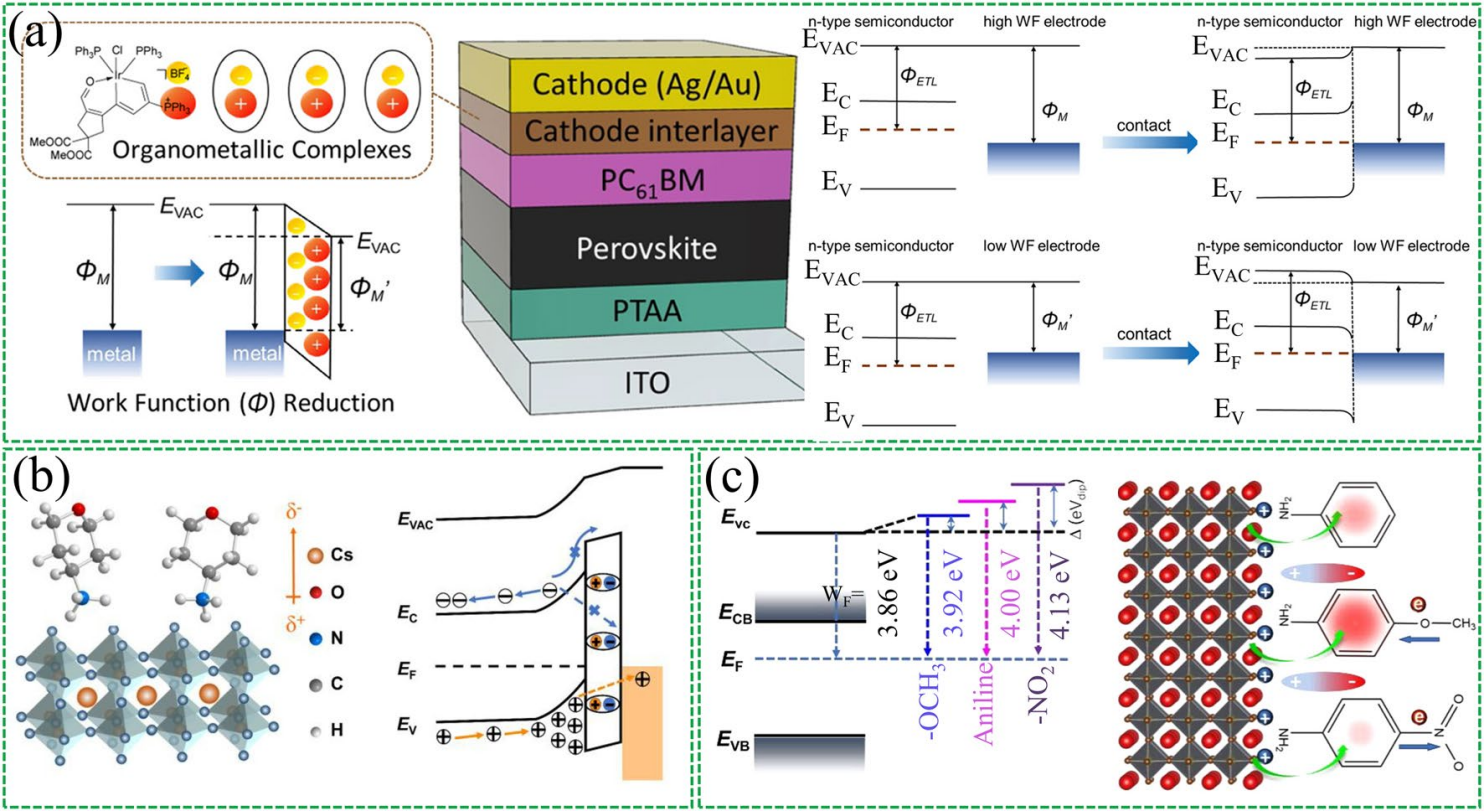
Insertion of interfacial dipole materials in the top metal electrode and the perovskite active layer. **a** Schematic diagram of the device using carbolong-derived organometallic composites as interfacial layers. Illustration of band bending in the condition of a high- work function and a low- work function metal as the cathode. Reproduced with permission from Ref. [110]. Copyright 2021, American Chemical Society. **b** Using ATHPBr dipole molecules to modulate the electronic state at the interface promotes hole extraction by improving the surface work function of CsPbI_2_Br. Reproduced with permission from Ref. [116]. Copyright 2022, Elsevier. **c** Schematic diagram of surface energy level reconstruction induced by different pendant groups of aniline-based molecules. Reproduced with permission from Ref. [117]. Copyright 2021, American Chemical Society

Wang et al. [110] recently reported on the use of carbolong-derived framework molecules as dipole interlayers at the cathode side of inverted PSCs (Fig. 5a). These molecules were found to reduce the work function of Au and Ag, leading to a rise in the vacuum energy level and improve electron collection efficiency. The S-shaped kink in the *J-V* curve was eliminated by reducing the cathode work function to a sufficiently low degree (4.0 eV). In addition to enhancing interfacial properties, the carbolong-derived complexes also improved environmental stability, with no efficiency loss observed after 4,080 h of storage. Similarly, Zhu et al. [111] found that silanes with -CF_3_ end groups induced a more efficient dipole effect and significantly enhanced device stability at the PCBM/Ag interface. Bao et al. [112] introduced isatin molecules and their derivatives into the back contact as a PCBM/Al cathode-modified interlayer, which improved carrier transport efficiency, eliminated hysteresis, and suppressed moisture-induced degradation of perovskite films. These findings suggest that the use of dipole interlayers is able to improve interfacial properties and stability simultaneously in PSCs.

On the other hand, metal electrodes are prone to corrosion and severe interfacial reactions, which has led to the exploration of alternative counter electrodes, such as carbon electrodes [113–115]. The use of carbon electrodes offers both cost advantages and inherent hydrophobic stability. To improve hole extraction. Land et al. [116] modified the interfacial electronic states of all-inorganic CsPbI_2_Br perovskite and carbon electrodes using 4-aminotetrahydropyran bromide (ATHPBr), which tunes the work function of the perovskite surface by forming an electric dipole layer at the interface (Fig. 5b). Finally, the device exhibits superior stability due to the suppressed interface reaction. The initial efficiency of 94% can still be maintained in the air environment for 1200 h. In a separate study, Tang et al. [117] designed a surface energy level reconstruction induced by a dipole moment (Fig. 5c), and investigated different side groups of aniline-based molecules, such as electron-withdrawing nitro (-NO_2_) or electron-donating methoxy (-OCH_3_). Their results demonstrated that only -OCH_3_-tailored molecules can perform efficient hole extraction, while -NO_2_ exhibits the opposite effect. These findings highlight the importance of side groups on interfacial dipole molecules for maximizing charge scavenging.

### Charge Transport Layer (CTL)/Perovskite Active Layer

The incorporation of charge transport layers (CTL) in TCOs and perovskite active layers remains a promising approach. By avoiding charge tunneling, CTL can be made thicker, thereby selectively facilitating the transport of charge carriers. The interface between the charge transport layer and the perovskite layer directly affects charge transporting, energy level matching, and device stability [63]. In addition, the lower interface also strongly affects the final morphology of the perovskite films in PSCs [26, 118]. The upper interface modification is crucial to the surface defects of perovskite films, such as ionic vacancies and unsaturated dangling bonds [119]. Among the modification strategies for these two interfaces, the interfacial dipole molecules stand out naturally due to their excellent properties.

#### Bottom CTL/Perovskite Active Layer

**Fig. 6:**
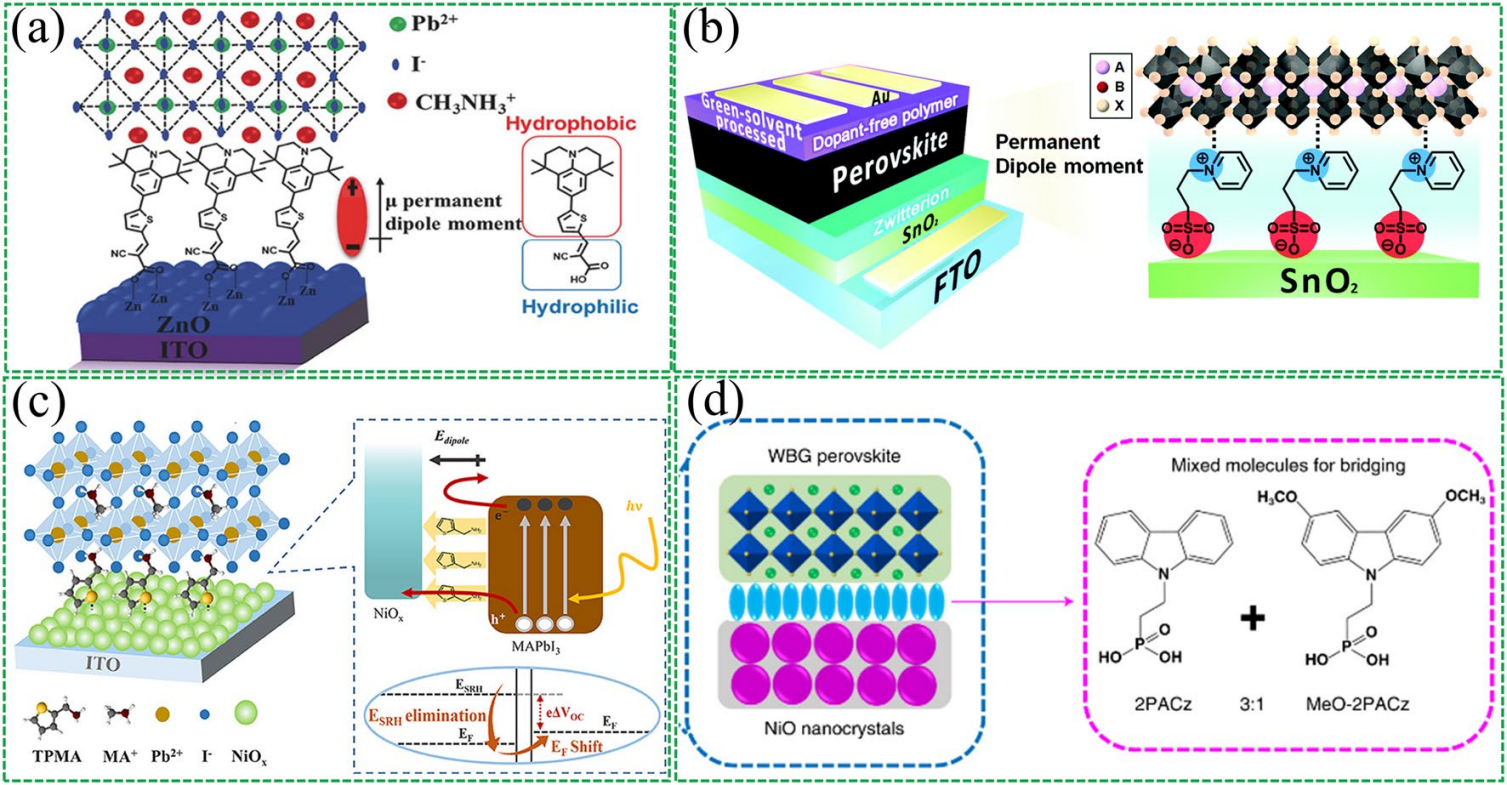
Insertion of interfacial dipole materials in the buried CTL and the perovskite active layer. **a** SAM-induced permanent dipole moment and energy levels at the ZnO/perovskite interface. Reproduced with permission from Ref. [122]. Copyright 2018, John Wiley and Sons. **b** Schematic diagram of the formation of interfacial dipoles by zwitterions on SnO_2_ layers. Reproduced with permission from Ref. [123]. Copyright 2018, Royal Society of Chemistry. **c** TPMA provides a moderate dipole moment directed toward the perovskite side, facilitating charge transport. Meanwhile, TPMA anchoring also passivated the defect states on the surfaces of NiO_x_ and MAPBI_3_. Reproduced with permission from Ref. [126]. Copyright 2022, American Chemical Society. **d** Device structure and molecular structures of bridging molecules. Reproduced with permission from Ref. [127]. Copyright 2022, Springer Nature

Zinc oxide (ZnO) can exhibit excellent electrical conductivity even when prepared at low temperatures. However, the surface properties of ZnO are not conducive to the growth of perovskite films with large grains [120, 121]. Accordingly, Jang et al. [122] synthesized a highly polar SAMs constructed at the interface of ZnO and perovskite active layer. In addition to improving the extraction of interface charges by interfacial dipole effect, SAMs also enhanced the hydrophobicity of the ZnO surface and thus improved the quality of the final perovskite film (Fig. 6a). SnO_2_ has recently become the most popular ETL for plate-structured PSCs. However, flat panel structure devices often suffer from hysteresis problems. Park et al. [123] employed zwitterion 3-(1-pyridinio)-1-propanesulfonate as a modifier at the SnO_2_/perovskite interface (Fig. 6b). The interfacial dipoles formed by zwitterions change the work function of SnO_2_, preventing reverse electron transfer and suppressing charge recombination. In addition, due to the passivation of the Pb-I anti-site traps of the perovskite, the photoelectric conversion efficiency and various stability of the device are improved, including long-term storage, light, heat and humidity conditions.

In inverted structures, it was found that the direction of the interfacial dipole moment leads to different consequences for PSCs. For example, it has been found that when dipole points to NiO_x_ inverted-structured PSCs, it will increase the *V*_OC_ of the device. While the opposite direction results in a loss of *V*_OC_ but an increase in short-circuit current (*J*_SC_) [124, 125]. Recently, our group [126] recently reported an application of a dipole material for the interface of NiO_x_ and perovskite layers (Fig. 6c). We design a moderately strong interfacial dipole moment to balance the contradiction between voltage and photocurrent enhancement. Specifically, we applied a 2-thienylmethylamine (TPMA) molecule with a moderate dipole moment, which was demonstrated by DFT calculations and KPFM tests to orient the dipole away from the NiO_x_ side. The *J*_SC_ is significantly improved to 23.72 mA cm^−2^ due to the efficient charge extraction capability provided by the dipole-induced additional driving force. Furthermore, TPMA provides a molecular passivation effect that compensates for the loss of *V*_OC_. The final device efficiency reaches 20.4%. Tan et al. [127] reported a hole-selective contact based on SAMs molecular bridging, in which hole-selective molecules were anchored on a low-temperature-processed NiO nanocrystalline film to alleviate interfacial recombination and to facilitate hole extraction in flexible PSCs (Fig. 6d). Specifically, a mixture of 2PACz and MeO-2PACz was used to adjust the energy-level alignment between NiO and the wide-bandgap perovskite, as the molecular dipole moments of 2PACz and MeO-2PACz are notably different (2.0 D and 0.2 D, respectively). Using this strategy, a flexible all-perovskite tandem solar cell with an efficiency of 24.7% was achieved.

#### Top CTL/perovskite Active Layer

**Fig. 7:**
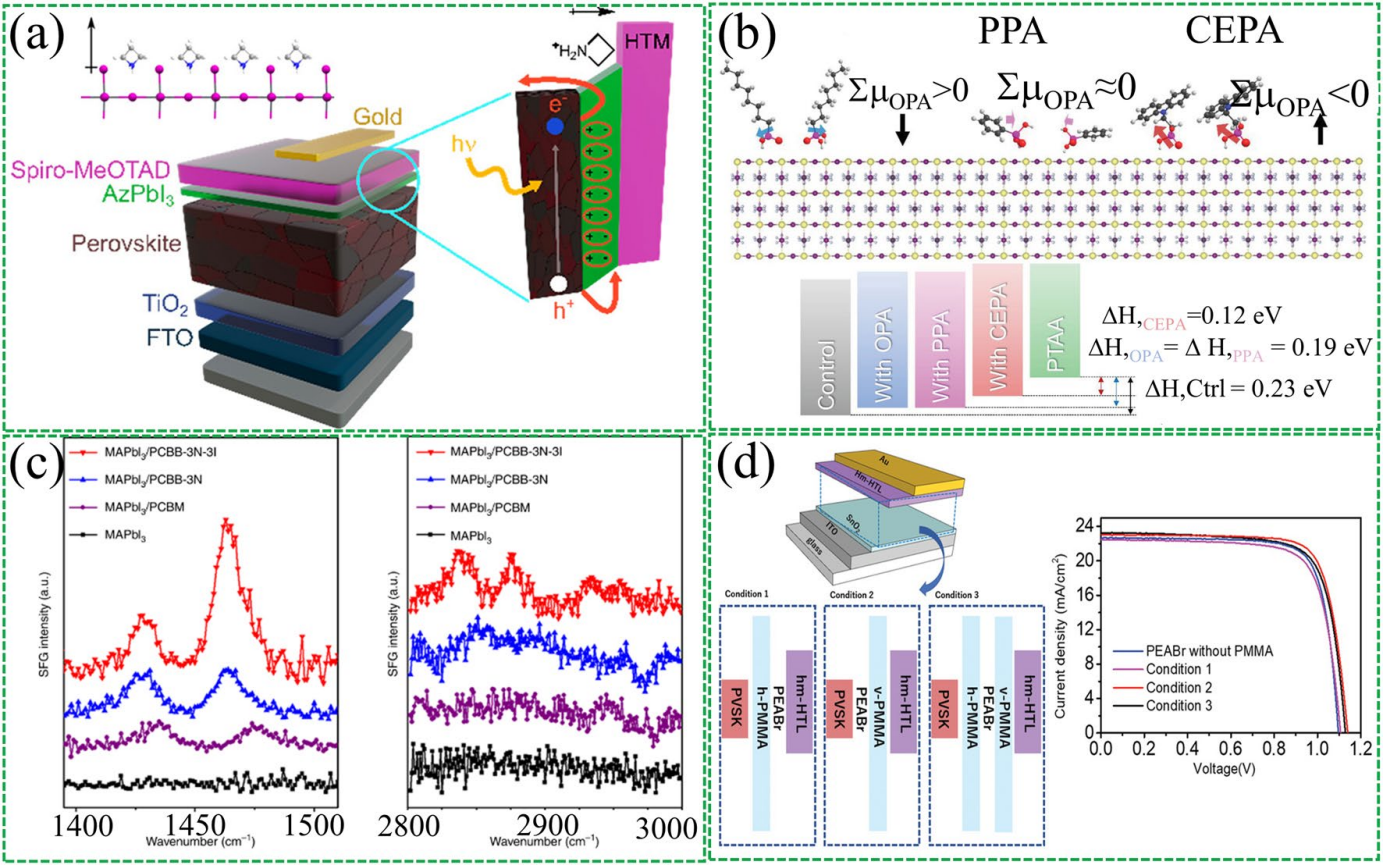
Insertion of interfacial dipole material in the top CTL and perovskite active layer. **a** Schematic illustration of the use of 1D (AzPbI_3_) as passivation and protective layers in PSCs, the formed dipole promotes hole transport. Reproduced with permission from Ref. [128]. Copyright 2020, American Chemical Society. **b** Energy-level alignment of the perovskite surface by controlling the functionality of Pas. Reproduced with permission from Ref. [129]. Copyright 2022, John Wiley and Sons. **c** SFG spectra of perovskites before and after treatment with PCBM, PCBB-3N-3I and PCBB-3N. Reproduced with permission from Ref. [69]. Copyright 2019, Springer Nature. **d** Schematic diagrams of three different structures and corresponding device efficiency diagrams. Reproduced with permission from Ref. [130]. Copyright 2021, John Wiley and Sons

The surface properties of perovskite films present a high density of defect states, which can result in under-coordinated lead atoms or halide anions that are detrimental to cell performance and stability. Therefore, surface defect passivation is critical. The treatment of the upper interface can passivate surface defects and reduce interfacial recombination. Moreover, the dipole layer formed can act as a protective thin layer to prevent perovskite film degradation from exposure to water and oxygen. A strategy proposed by Hagfeldt et al. [128] employed azetidinium lead iodide (AzPbI_3_) as a double passivation and protective layer to prepare efficient and stable PSCs (Fig. 7a). The ammonium groups in the Az cations form hydrogen bonds with the perovskite lattice, changing the work function of the perovskite surface. The final device efficiency is as high as 22%.

SAMs have shown promising results in PSCs due to their specific functional groups. Seo et al. [129] investigated how molecular design affects the dipole moment and interaction with hole transport materials (HTMs). These include [2-(9H-carbazol-9-yl) ethyl] phosphonic acid (CEPA), octylphosphonic acid (OPA), and phenylphosphonic acid (PPA). The study found that the apparent work function of perovskite is strongly influenced by the tail linking group (Fig. 7b), highlighting the importance of carefully considering molecular configurations to generate appropriate interfacial energy-level structures and ordered arrangements of dipole molecules. The strength of the interfacial dipole layer is not only on the dipole moment of the dipole molecule itself but also on the microscopic order of the dipole molecule at the interface. Zhang et al. [69] recently reported a study employing SFG spectroscopy to investigate the dipolar molecular order in fullerene-derived layers (PCBB-3N-3I, PCBB-3N, and PCBM) on perovskite surfaces. As shown in Fig. 7c, they suggest that the iodide in PCBB-3N-3I can bind to insufficiently coordinated Pb^2+^ to provide a driving force for molecular self-assembly at the interface, resulting in ordered packing and preferred molecular orientation.

The mechanism of surface treatment of three-dimensional perovskites (3D) with phenethylamine halides (PEAX) remains equivocal. A study was recently reported by Miyasaka's team [130]. They prevented direct contact at the perovskite/PEAX and PEAX/HTL interfaces by inserting a layer of poly (methyl methacrylate) (PMMA). Contrary to the widely observed phenomenon, the PCE value can be significantly increased when PEAX is not in contact with the bottom layer (perovskite) or top layer (HTL) (Fig. 7d). Through KPFM and UPS tests, it is found that the dipole electric field induced by PEAX at the interface is the main reason for the efficiency improvement, rather than the formation of low-dimensional perovskites. The combined use of PEAX/PMMA at the interface will further increase the dipole moment of PEAX. This in turn produces a high *V*_OC_ of 1.19 V and a PCE of 22.2%.

**Fig. 8:**
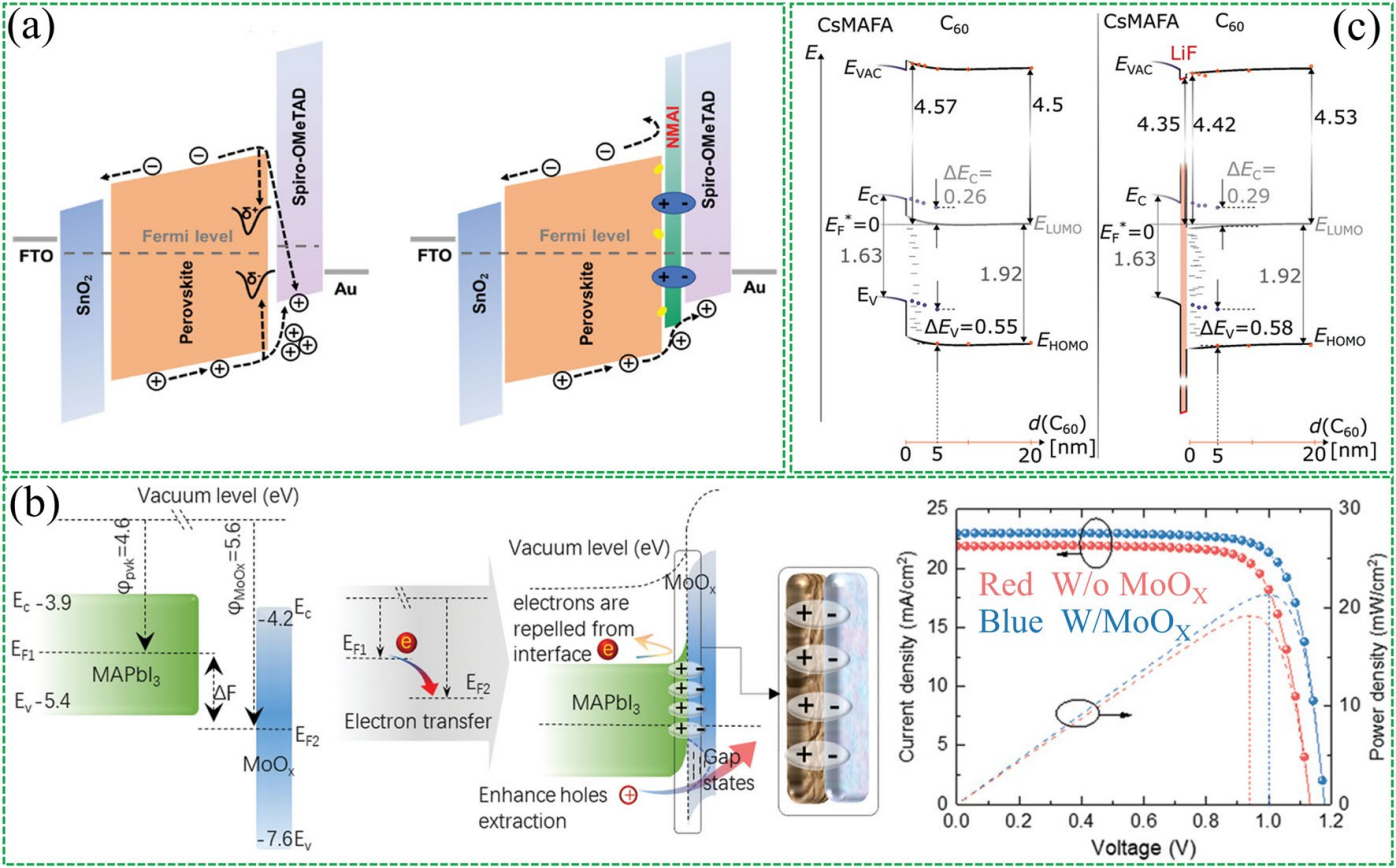
Interfacial dipole with field passivation effect. **a** Alignment of energy levels in PSCs without and with NMAI layers between perovskite and HTM. Reproduced with permission from Ref. [76]. Copyright 2020, John Wiley and Sons. **b** Schematic diagram of additional interfacial dipole formed by inserting MoO_X_ interlayer. Reproduced with permission from Ref. [19]. Copyright 2020, John Wiley and Sons. **c** Alignment of energy levels at the CsMAFA (with or without 1 nm LiF)/C60 interface. Reproduced with permission from Ref. [75]. Copyright 2022, John Wiley and Sons

Interfacial dipoles have been developed as FEP in PSCs, inspired by silicon solar cells. FEP reduces minority carrier recombination by separating electron–hole pairs from the recombination region [19]. Gao et al. [76] applied post-treatment on perovskite films with 1-naphthylmethylamine iodide (NMAI). Using this dielectric ammonium salt reduces defect-assisted recombination on the perovskite surface through chemical passivation. In addition, this ammonium salt induces energy level bending to provide an FEP to repel minority carrier recombination, thereby reducing charge accumulation (Fig. 8a). Finally, planar PSCs with *V*_OC_ up to 1.20 V were realized.

Yang et al. [19] conducted a more in-depth study on FEP. They incorporated a high work function MoO_X_ interlayer between the perovskite and hole transport layers. The interfacial work function difference will cause interfacial charge transfer and create a dipole field at the interface (Fig. 8b). This additional electric field can effectively separate photogenerated carriers and reduce carrier recombination. Numerical simulation results show that the dipole electric field will cause the splitting of electron/hole quasi-Fermi levels (*E*_fp_/*E*_fn_) to become larger, and the introduction of the interfacial dipole will significantly reduce the recombination rate of the rear interface. These results demonstrate that the interfacial polarization-induced dipole electric field enhances the built-in electric field of the device and reduces carrier recombination.

Recently Korte et al. [75] also investigated the FEP of LiF interlayers in PSCs. They found that adding a LiF interlayer between the perovskite layer and the fullerene C_60_ layer resulted in a significant increase in *V*_OC_. They determined the band alignment of the interface using ultrasensitive near-ultraviolet photoelectron spectroscopy (Fig. 8c). The results indicate that the increase in *V*_OC_ originates from the dipole effect at the interface and the possible existence of fixed positive charges. Both of these reduce the hole concentration at the perovskite/C_60_ interface. These findings demonstrate the potential of interfacial dipoles as FEP in improving the performance of PSCs.

### Synergistic Effects of Multiple Interfacial Dipole Layers

**Fig. 9:**
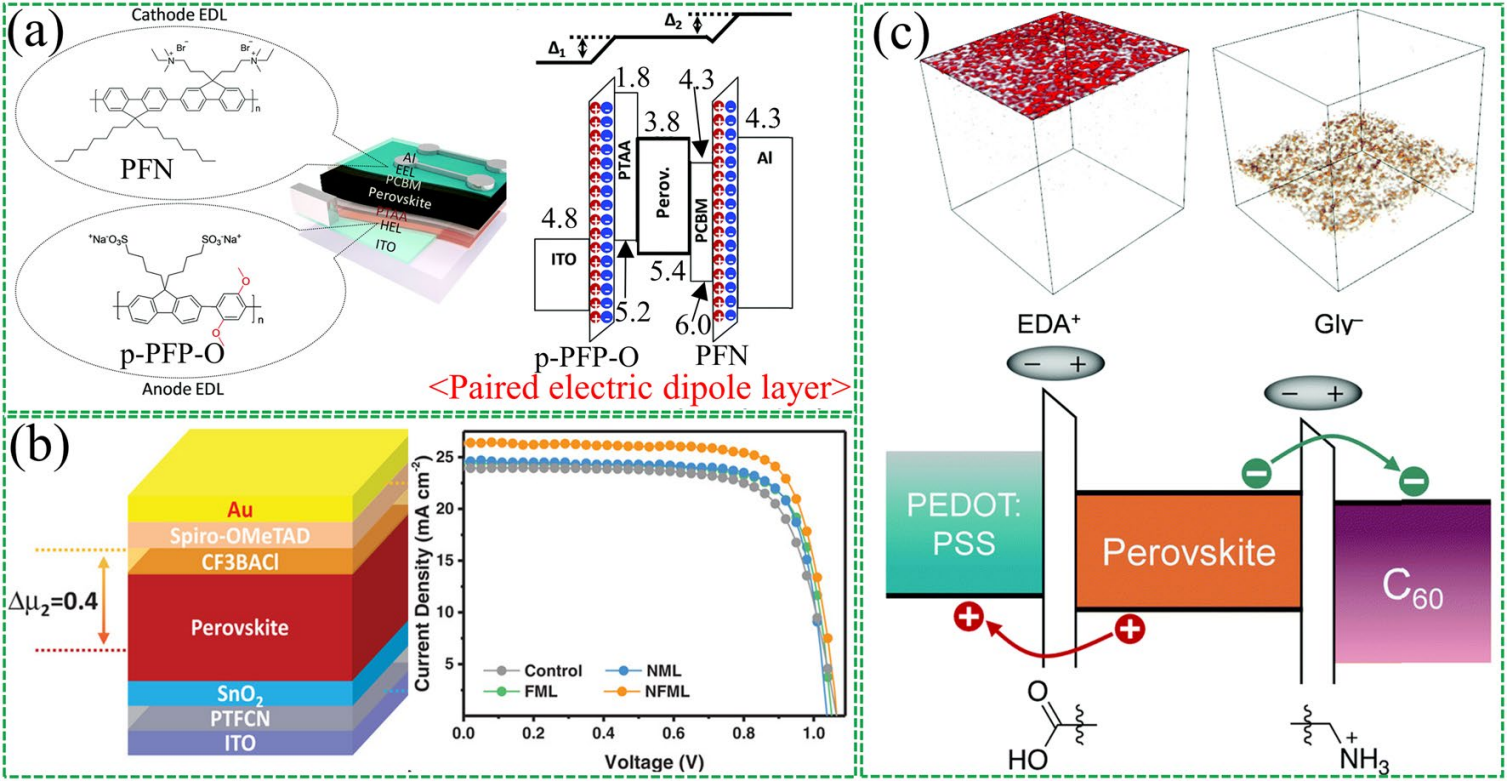
Synergistic effect of multiple interfacial dipole layers. **a** Schematic diagram of the chemical structure, device structure and energy-level structure of the paired electric dipole layer. Reproduced with permission from Ref. [134]. Copyright 2018, Royal Society of Chemistry. **b** Structure of NFML device and the J–V curves of the devices. Reproduced with permission from Ref. [56]. Copyright 2020, John Wiley and Sons. **c** Schematic diagram of the distribution of EDA^+^ and Gly^−^ ions in perovskite films and the resulting energy band changes. Reproduced with permission from Ref. [135]. Copyright 2022, Royal Society of Chemistry

During the research on polymer solar cells, it was found that strong power can be provided by applying an external reverse bias voltage to reduce recombination at defect sites [131–133]. The built-in electric field (*E*_in_) usually arises from the work function difference between the two electrodes in the device. However, for a single dipole layer, the *E*_in_ provided is necessarily limited. To address this limitation, Lee et al. [134] investigated the relationship between *E*_in_ and device performance changes in PSCs by introducing paired strongly electric dipole layers (EDLs) to tune the surface potentials of the hole and electron extraction layers (Fig. 9a). The extraction layers for the anode and cathode were p-doped poly(9,9-bis(4′-sulfonatobutyl)fluorene-alt-co-1,4-(2,5-dimethoxy)phenylene) (p-PFP-O) and PFN was used as the extraction layer for anode and cathode, respectively. The work function of p-PFP-O and PFN-modified ITO was found to be shifted by + 0.6 and -0.6 eV, respectively, by the Kelvin probe test. The results show that greatly enhanced *E*_in_ can be produced in the device by introducing paired layers of electric dipoles.

A similar double-dipole layer synergistic enhancement effect was reported by Feng et al. [56] Dipole molecules of dimethylamino (PTFCN) and trifluoride (CF_3_BACl) were introduced, respectively (Fig. 9b). It is found that PTFCN generates partially protonated nitrogen to form a permanent dipole moment, which effectively reduces the work function of the ITO substrate, thereby maximizing the *E*_in_ between the two electrodes. The trifluoride and amino groups of CF_3_BACl are covalently bonded through the benzene ring, which can provide a strong dipole moment to improve the work function of perovskite. Thus, the *E*_in_ on the upper and lower surfaces of the perovskite is increased. In addition, the hydrophobicity of the trifluoro groups on the surface of the perovskite film improves the long-term stability of the device. Recently, Wakamiya's team [135] modified the top and bottom of perovskite films with ethylenediammonium diiodide (EDAI_2_) and glycine hydrochloride (GlyHcl), respectively, in tin–lead hybrid PSCs (Fig. 9c). Both top and bottom were found to successfully induce surface dipoles and the direction of the dipole is consistent with the direction of *E*_in_, thus enhancing the *E*_in_ of the device.

These works highlight the significance of dipole molecules at internal interfaces in perovskite devices, with exciting synergistic effects observed when multiple interfacial dipole molecules are coupled. However, the different interfaces in devices exhibit diverse properties and functions, requiring unique design considerations, such as the orientation of dipole moments and specific active materials.

## Characterization of Interfacial Dipoles

Although interfacial dipoles can effectively tune the interfacial energy-level structures within devices, it is much more difficult to achieve direction characterization of their existence than the studies of bulk and surface properties of perovskite films or solar cell devices as due to the buried and ultrathin natures of interfacial dipoles [136–138]. Currently, interfacial dipole moment has been introduced to explain the various effects of the dipole interface material in PSCs, and the interaction model is developed to reveal the working mechanism of the local dipole moment [30]. However, due to the limitation of experimental characterization means, it is still difficult to directly observe the microscopic interaction processes at the interface of molecular layers. The dipole formation mechanism of the interfacial layer has not been well established yet, which makes the optimal design and promotion of interfacial dipole molecules in materials and devices difficult.

It is worth noting that the interfacial dipole moment strength is not only related to the dipole moment of the dipole material itself, but also closely related to the structure of the aggregated state of the dipole molecules. The completely random packing orientation may even cause the dipole moments of each molecule to cancel each other out. We believe that by developing more dependable and in situ characterization techniques for interfacial dipoles is essential to better comprehend and investigate their impact on the final device, especially by combining multiple techniques to resolve interfacial dipoles from multiple dimensions.

### Sum-Frequency Vibrational Spectroscopy (SFG)

SFG is a kind of second-order nonlinear optical spectroscopy, and the selection rule of the second-order nonlinear optical process only allows the generation of SFG signals from media without inversion symmetry [139]. Due to the breaking of interface inversion symmetry, SFG can be used to selectively study molecular information on surface and buried interfaces. It should be noted that this nonlinear spectral signal strongly depends on the quality of the pulsed laser beam used to generate the SFG signal. Furthermore, this is one of the most sensitive methods for studying molecular orientation at surface interfaces, including buried interfaces [140–143].

**Fig. 10:**
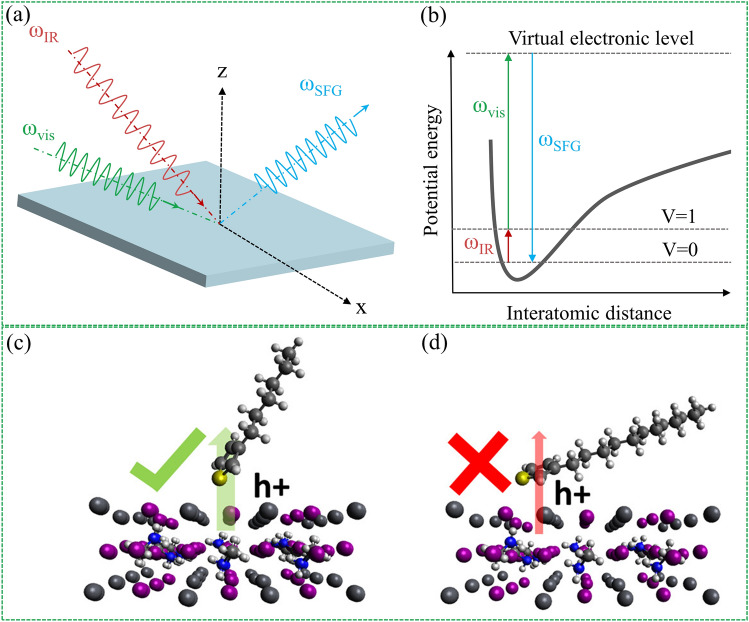
SFG technique for checking the orientation of interfacial dipoles. **a** SFG signal generation, and **b** SFG energy level diagram. Illustration of hole extraction ability of **c** PTs with shorter alkyl side chains and **d** PTs with a longer alkyl side chain at the HTL/perovskite interface. Reproduced with permission from Ref. [146]. Copyright 2017, American Chemical Society

Figure 10a illustrates the generation scheme for SFG signal generation. In a typical SFG experiment, a higher frequency but fixed visible light (Vis) and a tunable mid-infrared (IR) light are used as input beams. The bandwidth of the mid-infrared light determines the resolution of the SFG spectrometer. As long as the two beams of light can overlap at the same interface, the emitted SFG signal can be generated. Figure 10b illustrates the energy level diagram of the SFG signal generation process. The SFG signal is enhanced as the IR beam is tuned to the resonant frequency of the vibrational modes of the functional group at the interface. Therefore, the vibrational spectrum can be displayed as the characteristic fingerprint of the molecule. Therefore, SFG can also be used to identify interfacial functional groups. On the other hand, as mentioned earlier, the microscopic packing state of the interfacial dipole molecules is the key factor determining the interfacial dipole moment. Therefore, it is very important to obtain the orientation of dipolar molecular functional groups at the interface of PSCs. In fact, different SFG susceptibility tensor elements can be probed by collecting spectra at different polarization combinations (e.g., using different polarization combinations of the input and output beams), and thus the actual orientation of the molecules can be obtained [144, 145].

One important example is Chen et al. [146] successful correlation of the molecular orientation of the buried interface between perovskite and HTL using the SFG technique. Briefly, the molecular orientation of polythiophene (PT) depends on the alkyl side chain length (Fig. 10c-d). Since the dipole moment of the C = C stretching mode is more or less perpendicular to the thiophene ring, as a result, PT molecules with shorter alkyl side chains are erected at the interface, which is favorable for the extraction of interfacial holes, resulting in higher conversion efficiency.

### Near-edge X-ray Absorption Fine Structure (NEXAFS) Spectroscopy

NEXAFS spectroscopy has developed into a powerful technique for studying the orientation of molecules adsorbed on surfaces. The surface sensitivity of NEXAFS comes from tuning the X-ray beam to the absorption edge of elements present in the surface layer but not in the substrate, and the technique has distinct advantages for monolayer or sub-monolayer samples. Recently, the application of NEXAFS spectroscopy has been extended to thin films, especially for dipole layer materials at the interface, relying on the limited electron mean free path in solids, still achieving high surface sensitivity using electron yield detection.

**Fig. 11:**
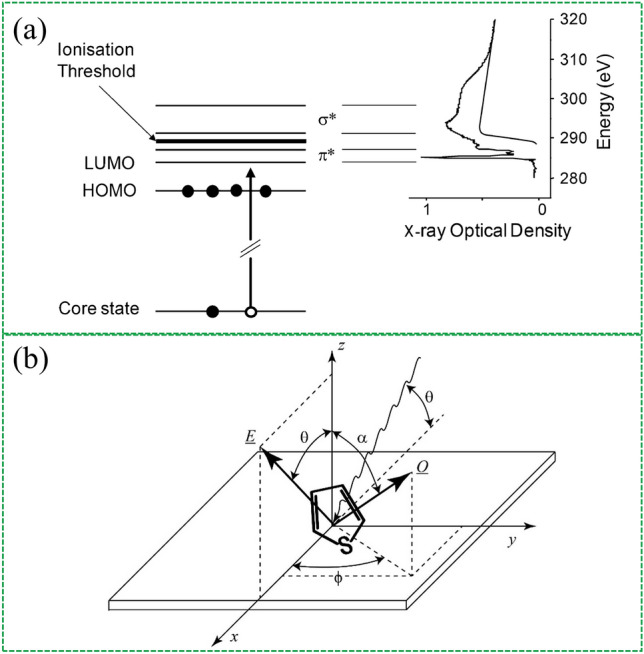
NEXAFS spectroscopy for examining interfacial molecular orientation. **a** Schematic energy diagram showing possible electronic transitions from a core state to anti-bonding and continuum states following absorption of an X-ray by a conjugated polymer, and the associated NEXAFS spectrum. For simplicity, Rydberg states are not shown. **b** Geometry of an angle-resolved NEXAFS spectroscopy experiment to determine the molecular orientation of a thiophene ring for the plane of the substrate. Reproduced with permission from Ref. [147]. Copyright 2016, Elsevier

Since each element has a different characteristic X-ray absorption edge that corresponds to the different energies required to ionize different elements by removing electrons from different core levels. Near the absorption edge, there are additional non-ionizing resonance transitions from the relevant core levels to the associated unoccupied electronic states of the final state π* and σ* antibonding orbitals. The NEXAFS spectra of molecules are often characterized by resonant transitions from core states to antibonding molecular orbitals and continuum states above the vacuum level (Fig. 11a**)** [147]. By measuring NEXAFS spectra of conjugated polymers at different incident polarizations, the change in transition resonance intensity can be used to determine the orientation of the transition dipole moment. When the orientation of the transition dipole moment relative to the molecular structure is known, the observed dichroism of a particular transition can be used to determine the molecular orientation (Fig. 11b). This review focuses on the properties of the interfacial dipole, and readers interested in more details of the NEXAFS spectra can refer to the previous report [148–151]. For example, Arramel et al. [152] utilized angle-dependent NEXAFS spectroscopy to study the orientations of the interfacial dipole molecules that kinetically blocked heptazethrene triisopropylsilyl ethynylene (HZ-TIPS) on CsPbBr_3_.

### Kelvin Probe Force Microscopy (KPFM)

**Fig. 12:**
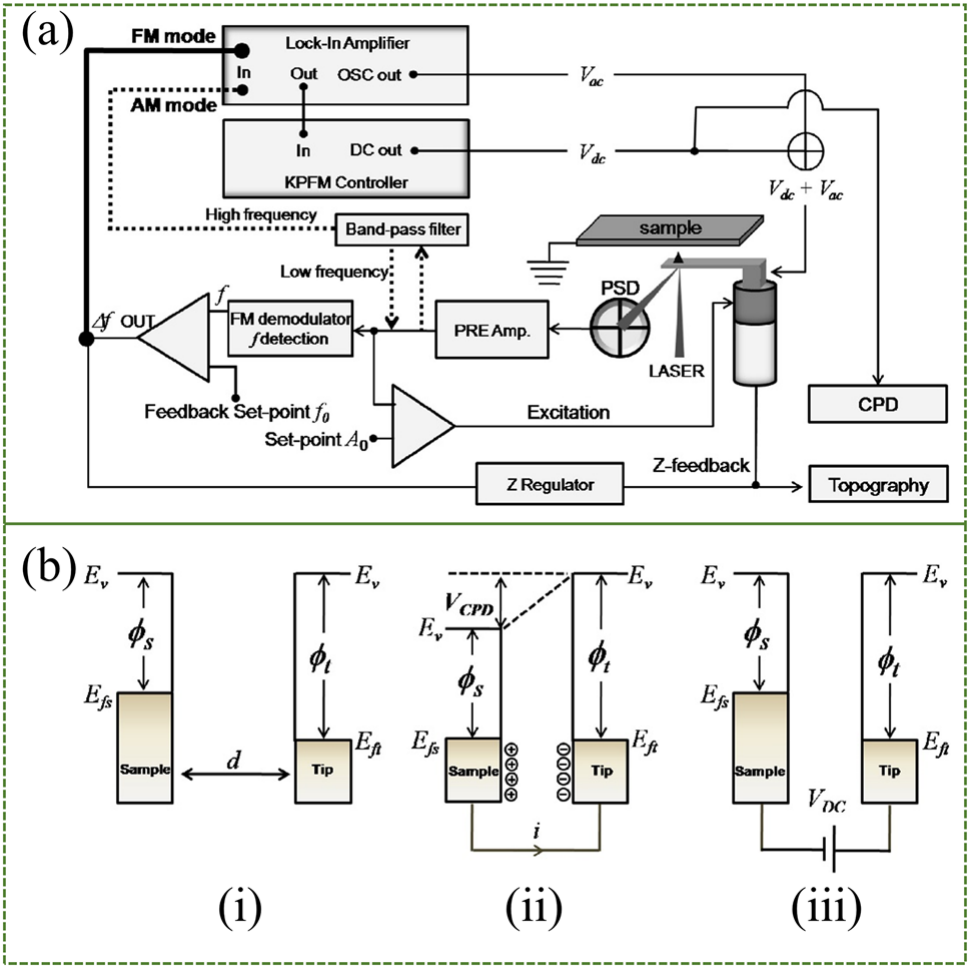
KPFM technique for measuring interfacial dipoles. **a** Schematic diagram of KPFM system showing AM and FM mode. **b**The electron energy levels of the sample and tip in the three cases. Panel (i) shows before contact, (ii) during electrical contact, (iii) with an external bias V_DC_ applied between the tip and the sample to offset the CPD. Reproduced with permission from Ref. [154]. Copyright 2011, Elsevier

As a non-destructive technique, KPFM provides precise surface information at the microscopic scale and is an important method to directly measure interfacial dipoles. When two substances come into electrical contact, electrons immediately flow from the side with the smaller work function to the side with the larger work function, until the two substances reach the same electrochemical potential to establish equilibrium. On the other hand, the surface work function depends on the energy difference between the vacuum and the Fermi level [153]. In the KPFM test system, electrostatic forces are generated due to the Fermi level difference between the tip and the sample. The contact potential difference (CPD) of the interface is obtained by extracting the electrostatic force. This experimental setup is illustrated in Fig. 12a, where the dashed and thick straight lines represent the KPFM configuration for amplitude modulation (AM) and frequency modulation (FM) modes [154]. In general, CPD is highly material-dependent and is related to the work function of the pure material and the additional surface dipole moment. Once we have obtained the work function of the tip (*Ф*_tip_), the work function of the sample (*Ф*_sample_) to be tested can be obtained by a simple formula:

4 $$  \phi_{{{\text{sample}}}} =\phi_{{{\text{tip}}}} - {\text{e}}{ \times }{\text{CPD}} $$

where e is the electron charge. In a KPFM experiment, topography measurements are performed in the first pass using mechanically actuated tapping mode, as followed by raising the tip height (Δz) in the second pass while applying alternating voltage (*V*_AC_), in which the potential difference between the probe and sample thus causes mechanical oscillations of the probe, and is then counteracted by the applied direct voltage (*V*_DC_) through a potential feedback loop. Figure 12b depicts the energy level diagrams for three different situations. When the sample and the tip are in contact, electron transfer occurs until the Fermi level is the same. When an external bias V_DC_ equal to the CPD is applied between the sample and the tip, the tip and the sample surface charge are eliminated. Hence *V*_DC_ = CPD.

The dipole layer at the interface can be considered as a two-dimensional set of dipoles whose orientations are inferred from the positive signal relative to the change in the surface potential of the tip. For example, Russell et al. [155] reported inserting C_60_-N as an interlayer between the Ag electrode and ETL, using KPFM to visualize the existence of a negative interfacial dipole between Ag and C_60_-N, improving the electron extraction rate at the interface, and finally realizing the device efficiency improvement.

**Fig. 13:**
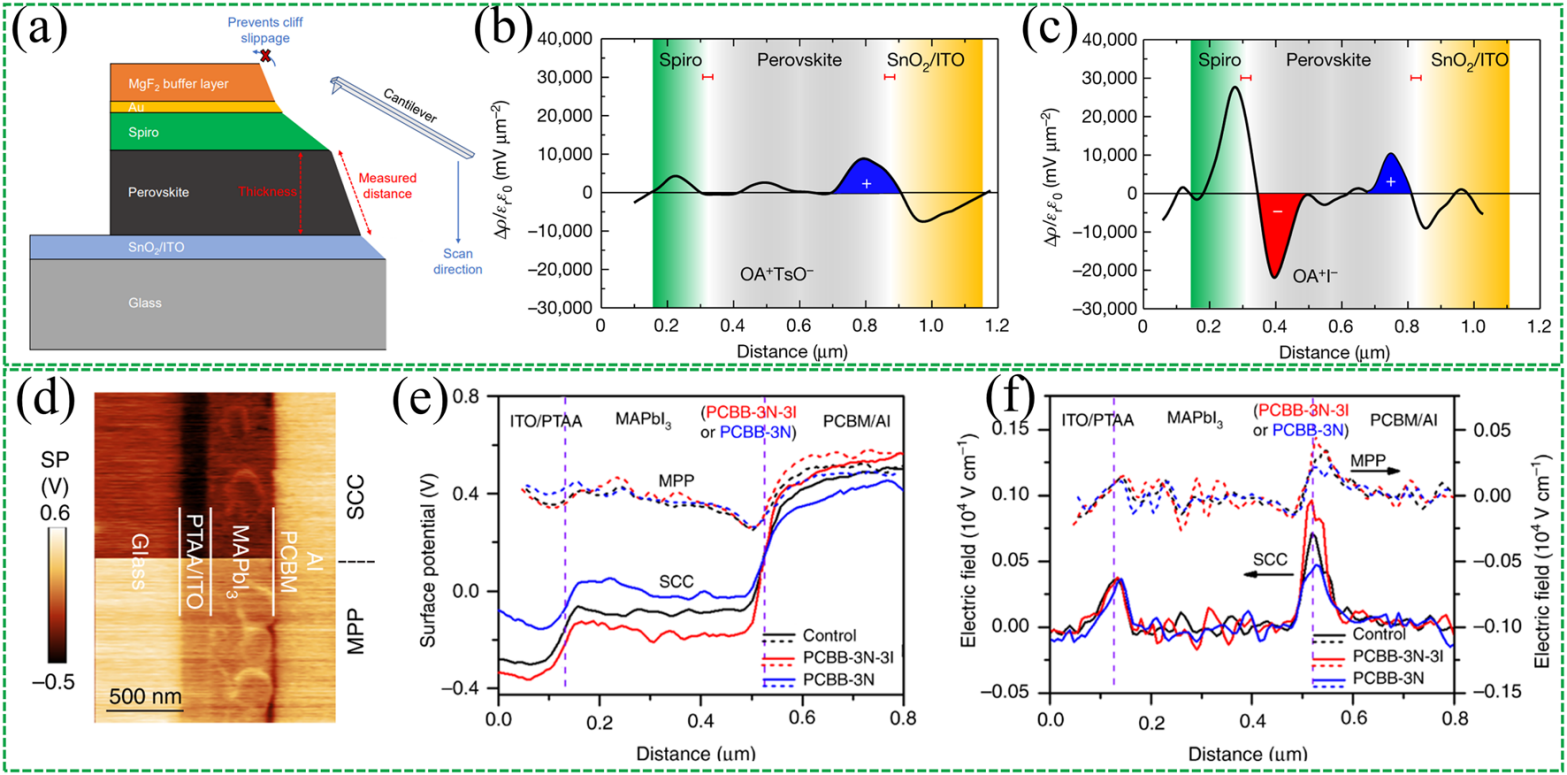
Cross-sectional KPFM schematic. **a** Schematic illustrating the KPFM measurement setup and planar device structure of ITO/SnO_2_/perovskite/spiro-OMeTAD/Au. Charge density distribution profiles of** b** the complete OATsO-treated and** c** OAI-treated device cross sections measured by cross-sectional KPFM. ρ, charge density; ε_0,_ vacuum permittivity; ε_*r*_, relative permittivity. The devices were illuminated under the OC condition. The red error bars demarcate the estimated spatial resolution of about 30 nm. Reproduced with permission from Ref. [157]. Copyright 2022, Springer Nature. **d** SKPM-measured SP image in SCC (top half) and MPP (bottom half). **e** SP depth profiles and **f** corresponding electric field distribution of the control device (black), PCBB-3N-3I device (red), and PCBB-3N device (blue) in SCC (solid line) and MPP (dashed line)). Reproduced with permission from Ref. [69]. Copyright 2019, Springer Nature

Due to the spatially confined and ultrathin natures of interfacial dipoles, detecting the dynamic changes of dipoles in the functional devices is extremely challenging. Fortunately, KPFM provides excellent lateral spatial resolution (< 50 nm) and energy resolution (~ 10 meV) [156], which is a powerful tool for detecting dipoles at the buried interfaces of cross-sectional samples, allowing visualization of the dynamic changes in potential displacement of the cross-sectional sample in operating devices. It should be noted that the cross-sectional surface should not be further processed (e.g., polished), as long as effective KPFM imaging can be performed. Because KPFM measurements are sensitive to surface height, any excessive modification may cause artifacts. Yang et al. [157] measured cross-sectional KPFM under open-circuit (OC) conditions and illumination. The top MgF_2_ buffer layer was used to mitigate the uncontrollability of the mechanical cutting process (Fig. 13a). The charge accumulation across the interface was intuitively visualized (Fig. 13b-c), which corresponded to different post-processing scenarios of dipole molecules. Zhang et al. [69] conducted cross-sectional KPFM measurements to detect surface potential (Fig. 13d). Owing to the dipole-induced field, the drop of surface potential at the perovskite/PCBM interface after treatment with dipole materials resulted in significant enhancement of the local electric field (Fig. 13e-f).

### Ultraviolet Photoelectron Spectroscopy (UPS)

**Fig. 14:**
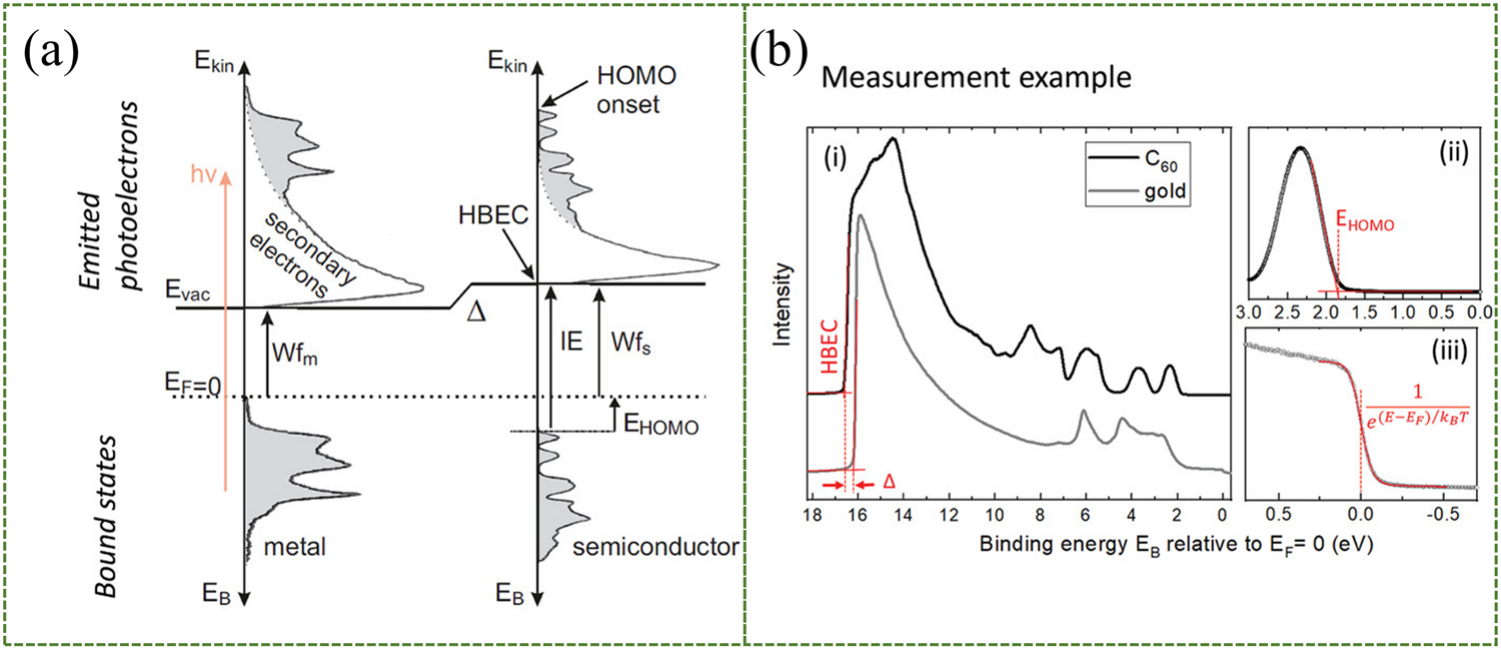
UPS technology for measuring interfacial dipoles. Principle of dipole electric field measurement by UPS. **a** The ionization process of metals as well as organic semiconductors. **b** Examples of UPS measurements of metal (gold) and organic semiconductor (fullerene C_60_) deposition on gold substrates. An offset is found, denoted as the interfacial dipole moment Δ. Reproduced with permission from Ref. [158]. Copyright 2021, John Wiley and Sons

The working principle of ultraviolet photoelectron spectroscopy is based on the excitation of photons to the sample to be measured. If the excitation energy is suitable enough, it will cause the release of photoelectrons with certain kinetic energy (*E*_kin_). It can overcome the vacuum level *E*_vac_. Without considering inelastic scattering, by the conservation of the total energy, the binding energy (*E*_B_) of the electron in the initial state can be calculated as:

5 $$ E_{B} = hv - E_{{{\text{kin}}}} $$

where *hν* is the incident ultraviolet photon energy. When conducting UPS experiments, attention must be paid to the sensitivity of electrons as a means of detection. This is because the mean free path of the generated photoelectrons is small, so the UPS measurements are only for a few nanometers at the surface. This special restriction is a very useful property for questionnaire interfaces. For example, the ionization process of classical metals and organic semiconductors is shown in Fig. 14a. The electron energy at the secondary electron cutoff or high binding energy cutoff (*E*_*cutoff*_) is just enough to cross the vacuum level. Therefore, the sample work function information is provided here [158]:

6 $$ W_{f} = hv - E_{{{\text{cutoff}}}} $$

when dipole molecules are deposited at the interface, the dipole moment can be extracted from the offset Δ of the *E*_*cutoff*_ of the samples before and after deposition. Also, the direction of the dipole is obtained from the direction of Δ. Examples of UPS measurements of metals (gold) and organic semiconductors (fullerene C_60_) deposited on substrates are shown in Fig. 14b.

Also, for example in the recent report by Tseng et al., an interfacial dipole of 0.3 eV was found between the ITO electrode and the NiO_X_ layer far from the active layer by UPS measurements [159]. The dipole significantly enhanced hole extraction at the ITO/MAPbI_3_ interface.

### Other Methods

For the confirmation of the interfacial dipole moment, it is mainly to obtain its surface potential or work function, so other techniques can also possibly be used to measure it. Examples include photoelectron spectroscopy (PES), electron beam induced current (EBIC), and scanning electron microscopy (SEM). The test principle of KP is similar to that of KPFM, but KP is an averaging method and can only obtain the CPD value of the entire sample area. PES works by measuring the energy of photo excited electrons emitted from the surface of a sample, which can be used to determine the electronic band structure of the sample. The original work of EBIC is to measure the current generated by electron–hole pairs, so it is only suitable for semiconductor samples. SEM resolves the local electronic structure on the surface of semiconductor samples by measuring EBIC. Valence band (VB) movement can also be monitored electrochemically, such as by cyclic voltammetry (CV) [160]. In addition, the researchers have recently used constant final state yield spectroscopy (CFSYS) to study the dipole effect at the interface of perovskite and C_60_. This ultra-sensitive near-UV photoelectron spectroscopy technique can probe anomalously high detection depths of 5 ~ 10 nm. By directly observing the Fermi edge in the CFSYS spectra, more accurate interfacial energetic offsets can be evaluated [75]. Finally, the orientation and strength of the interfacial dipoles can also be indirectly inferred from knowledge of the molecular structure and arrangement.

## Summary and Outlook

In summary, we analyze the fundamental properties of electric dipoles and their specific roles in PSCs. We systematically review the recent advances in the research and development of dipole materials at several key interfaces relevant to the carrier extraction and performance stability of PSCs. To further elucidate the effects of electric dipoles in modifying the device interfaces, we also highlight numerous analytical techniques to render unambiguous characterizations of the dipole effects. Based on the aforementioned works, several conclusions can be deduced from the abovementioned works:(i) The field-effect properties of the interfacial dipole can easily tune the apparent work function of the substrate and enhance the actual built-in electric field of the device, thereby facilitating carrier transport across the interface. (ii) Functional groups of dipole molecules provide additional chemical passivation effects to the interfaces while greatly inhibiting ion migration across the interfaces, thus notably improving device stability under operational conditions. (iii) Site-specificity of the electric dipoles widens the functional versatility at the device interfaces. In addition, simultaneous enhancements of multiple device interfaces with specifically tailored dipole layers will lead to synergistic performance enhancements.

Although dipole engineering has shown promising advantages in improving energy-level alignment, charge extraction, defect passivation, and stability of PSCs from the device interfaces, unresolved research aspects nevertheless still exist that demand elucidation to further mature the applicability of electric dipoles in PSCs, where the future research directions are outlined as followed

1. **Molecular configuration designs of dipole materials.** Currently, there is still a lack of clear guidelines in the process of seeking to optimize the screening of interfacial dipole materials from a molecular configuration perspective. In general, ideal candidate materials for interfacial dipoles should possess both functional diversity and ease of processing. For example, it needs to create an ideal energy level arrangement to facilitate charge transfer and ideally also passivate multiple types of defects in the perovskite layer to mitigate ion migration and enhance the operational stability of the device. In addition, cost advantage and environmental friendliness also need to be considered to address future commercialization challenges. There are still great opportunities for future dipole molecular configuration design. (1) Molecular designs of dipole materials: The structure and chemical properties of dipole molecules, including their polarity, dipole moment, orientation, steric hindrance, electronegativity, length, and size of the fatty-acid chain/aromatic ring, should all be considered when designing dipole materials with highly ordered orientation and dipole moment. (2) Interface affinity: Dipole interface materials need to form stable interfaces with other layers, so interface affinity should be considered in molecular design. Interface affinity is influenced by many factors, including the electronic structure, chemical structure, and surface activity of the molecules. (3) Stability of dipole materials: Interfacial dipole materials need to have strong thermodynamic stability and low chemical reactivity. Most research in the field is currently based on organic dipole materials, and future research should also focus on inorganic materials with higher stability, such as back-field passivation techniques developed for silicon batteries. (4) Machine learning-assisted design: Currently, most dipole materials are discovered through trial and error, which requires long-term experimental processes that are time-consuming and technically challenging [161, 162]. In future, machine learning can be used to predict the electronic structure, stability, and interface affinity of molecules, accelerating the screening of the most promising molecules and guiding molecular configuration design. For example, it is known that hydrophobic functional groups such as long alkyl chains or C-F bonds can improve the long-term stability of the device. Otherwise, functional groups containing lone pairs of electrons can cause dipole effects by electron cloud rearrangement.
2. **Decoupling electric-field and chemical effects of the dipole materials.** Considering the presence of terminal functional groups with chemical passivation effects in the dipole molecules, one may expect that the applied dipole molecules can result in synergistic increments of *V*_OC_ by both chemical passivation and intrinsic electric-field effects, with similar ideas being pointed out in some recent works [76, 163]. Yet, the respective contributions of these two mechanisms in governing the performance of PSC devices are often puzzling. Theoretical modeling of the changes in carrier concentrations, interfacial band bending effects, and the resultant quasi Fermi-level splitting by the chemical and electric-field effects, can deconvolute the, respectively, enhanced magnitudes of the PV performance parameters.
3. **Deterministic and reliable characterization of electric dipoles.** So far, KPFM and UPS are the mostly adopted analytical methods in the characterization of interfacial dipoles after the whole PSCs are completely assembled. These methods either require device’s cross section as the specimens or etched surfaces to expose the functionalized interfaces, which are often destructive to the ultrathin nature of electric dipole materials. Therefore, characterizations with in-situ manners should be properly developed and applied to investigate the anchoring and effects of electric dipoles as they are delivered to the device interfaces. For example, in-situ KPFM, surface photovoltage (SPV), UPS and/or XPS measurements are desirable to display the real-time variation of chemical environments and energy states upon the material surface being treated.
4. **The practical challenges of future commercialization**. Although the PSCs have achieved remarkable performance, future commercialization still faces challenges. Previous works regarding dipoles have mainly focused on improving performance of PSC devices, with most neglecting the photothermal stabilities of interfacial dipole materials to meet future commercialization demands. For instance, some dipole materials, especially organic small-molecule SAMs, are prone to evaporate at high temperatures. Standards matching the International Summit on Organic Photovoltaic Stability (ISOS) protocol should be established to guide research on interfacial dipole materials. Furthermore, the introduction of interfacial dipole materials is an additional process that will inevitably increase the overall cost of equipment. Therefore, developing low-cost, multifunctional interfacial dipole materials is critical for commercialization. Lastly, most research reports are based on laboratory-scale small-area PSCs. It remains largely unexplored whether dipole molecular interfacial processing can still exhibit remarkable effects in large-area production. In particular, there are still challenges in the process of amplifying dipole molecules during production. Future research trends are to develop large-scale production processes compatible with production lines, such as screen printing, blade coating, and thermal evaporation.
