# Author Correction: Recent Progress in Interfacial Dipole Engineering for Perovskite Solar Cells

**DOI:** 10.1007/s40820-024-01402-8

**Published:** 2024-05-02

**Authors:** Yinyi Ma, Jue Gong, Peng Zeng, Mingzhen Liu

**Affiliations:** 1 School of Materials and Energy, University of Electronic Science and Technology of China, Chengdu, 611731 People’s Republic of China; 2 State Key Laboratory Electronic Thin Film and Integrated Devices, University of Electronic Science and Technology of China, Chengdu, 611731 People’s Republic of China

**Correction to: Nano-Micro Lett. (2023) 15:173** 10.1007/s40820-023-01131-4.

In the version of this Article originally published online, there was an error in the schematics of Figures 2b and 2c. These errors have now been corrected in the original article.
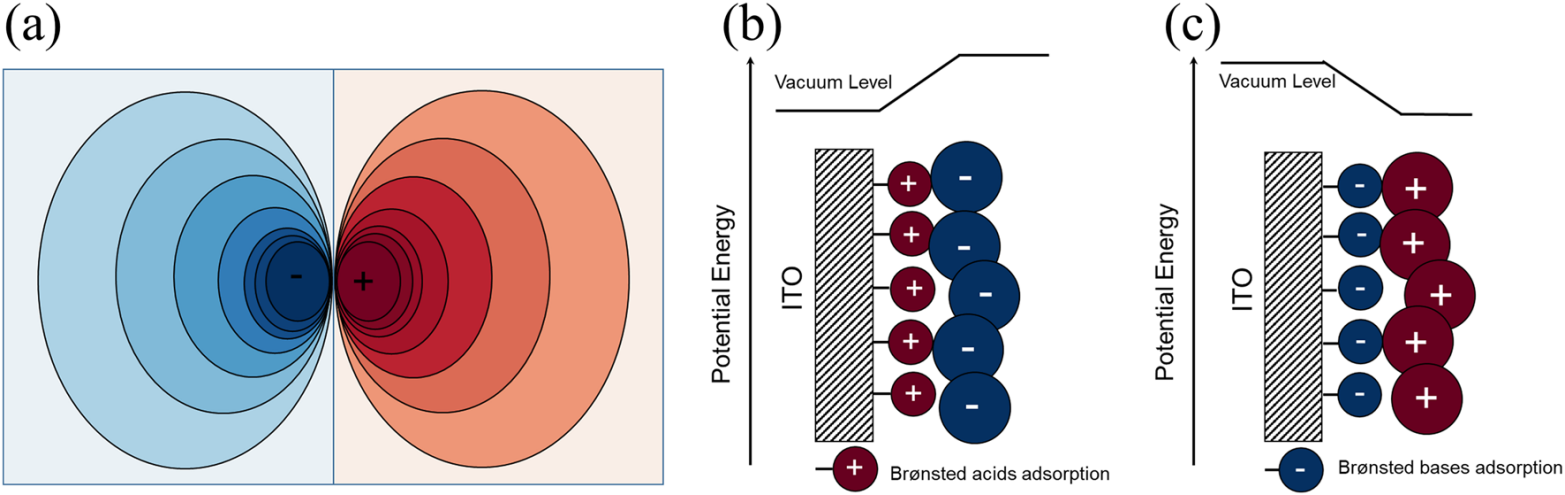


Original and corrected of Figures 2b and 2c.

